# A multiscale signaling–biophysical framework reveals mechanisms of macrophage-mediated RBC clearance in sickle cell and Gaucher disease

**DOI:** 10.1093/pnasnexus/pgag348

**Published:** 2026-10-06

**Authors:** Zhaojie Chai, Nazanin Ahmadi Daryakenari, George E Karniadakis

**Affiliations:** Division of Applied Mathematics, Brown University, Providence, RI, USA; Center for Biomedical Engineering, School of Engineering, Brown University, Providence, RI, USA; Division of Applied Mathematics, Brown University, Providence, RI, USA

**Keywords:** red blood cells, phagocytosis, physics-informed networks, multiscale modeling

## Abstract

Red blood cell (RBC) clearance by macrophages maintains blood homeostasis and is dysregulated in the hemolytic disorder sickle cell disease (SCD) and the lysosomal storage disorder Gaucher disease (GD), where biophysical and biochemical alterations promote premature phagocytosis. We develop a multiscale hybrid modeling framework integrating signaling dynamics, biophysical simulations, and machine learning to investigate the mechanisms governing RBC phagocytosis in these diseases. Our approach couples a systems biology model of macrophage–RBC signaling with dissipative particle dynamics (DPD) simulations of molecular diffusion and membrane interactions, and leverages Physics-Informed Neural Networks (PINNs) for simultaneous parameter inference, hidden-state reconstruction, and integration of multiscale mechanistic constraints. The DPD framework provides mechanistic insight into antibody diffusion, receptor engagement, and membrane-level interactions during macrophage–RBC contact, generating spatially resolved trajectories of CD47–SIRP*α* signaling and antibody–receptor binding that serve as intermediate observables constraining the signaling model. The model accurately captures differential phagocytic responses between healthy and altered RBCs, revealing diminished inhibitory signaling and changes in SHP1-mediated pathways in both SCD and GD. Identifiability analysis combining Fisher Information Matrix diagnostics and profile likelihood confirms that parameters governing the CD47–SIRP*α*–SHP1 axis are among the most robustly recoverable, and simulations of therapeutic perturbations with anti-SIRP*α* antibodies demonstrate modulation of engulfment outcomes. We further employ Physics-Informed Kolmogorov-Arnold Networks (PIKANs) as an alternative to standard PINNs, demonstrating improved robustness under noise and sampling variability. More broadly, our multiscale platform linking biophysical simulation with systems-level inference is generalizable, offering mechanistic insights and computational tools for therapeutic exploration in diseases involving dysregulated phagocytosis.

Significance statement Red blood cells are normally removed from circulation by macrophages through tightly regulated signals. In sickle cell disease and Gaucher disease, this process becomes abnormal, contributing to anemia and other complications. However, the mechanisms linking antibody binding and receptor engagement to macrophage decision-making remain poorly understood. Here, we develop a multiscale computational framework that combines particle-based biophysical simulations, systems biology models, and physics-informed machine learning. This approach couples antibody diffusion and receptor engagement at the macrophage–RBC interface with intracellular signaling dynamics, providing a quantitative framework to interpret how perturbations of the CD47–SIRP*α* inhibitory checkpoint reshape phagocytic decisions in disease. The framework provides a predictive platform for studying immune clearance and may guide therapies targeting red blood cell–macrophage interactions.

## Introduction

Red blood cells (RBCs) circulate for about 120 days, after which aged or damaged RBCs are removed by macrophages in the spleen to maintain healthy blood cell populations. This clearance process relies on a balance of “self” signals that prevent unwarranted phagocytosis and “eat-me” signals that mark defective RBCs for removal. A well-known inhibitory signal is the interaction between RBC surface protein CD47 and macrophage SIRP*α*, which engages a “don’t eat me” pathway (1–4). Conversely, exposure of phosphatidylserine (PS) on RBCs or opsonization of RBCs can trigger prophagocytic pathways via macrophage Fc*γ* receptors and other receptors (5, 6). These molecular interactions converge on the macrophage cytoskeletal machinery. For instance, SIRP*α* signaling recruits the phosphatase SHP1, which in turn modulates myosin IIa activity required for engulfment (7).

In sickle cell disease and Gaucher disease, pathological alterations in RBC properties upset this balance. Sickle cell disease (SCD), caused by a hemoglobin mutation, produces misshapen and rigid RBCs with altered membrane protein display. These sickle RBCs tend to become trapped in the spleen and are cleared more rapidly than normal cells, which contributes to anemia and acute splenic sequestration crises (8). Studies by Khandelwal et al. (9) indicated that sickle cells may have reduced effective CD47-SIRP*α* signaling, diminishing their ability to convey a “self” signal and thereby permitting higher phagocytic uptake. In GD, deficiency of glucocerebrosidase leads to systemic accumulation of glucosylceramide, including altered lipid composition of RBC membranes, as shown by Franco et al. (10) and Dupuis et al. (11). Gaucher RBCs exhibit abnormal biomechanics, including increased rigidity and greater aggregation propensity, together with altered membrane composition, which affects their deformability and surface signaling, as described by Adar and colleagues (12, 13). These alterations are thought to promote splenic trapping and phagocytosis of Gaucher RBCs, contributing to anemia and splenomegaly in patients (14).

Despite extensive characterization of RBC deformability and adhesion in both SCD and GD, the precise multiscale mechanisms by which these alterations affect macrophage signaling and engulfment, that is, erythrophagocytosis, remain incompletely understood. To address this knowledge gap, we developed an integrative computational model that merges biochemical signaling pathways with biophysical cell interaction dynamics. Because experimental in vitro measurements alone cannot fully resolve the spatial and temporal dynamics of macrophage–RBC interactions, we employ complementary computational approaches.

In our framework, a system of ordinary differential equations (ODEs) captures the key signaling network involved in RBC phagocytosis, including the dynamics of the CD47-SIRP*α*-SHP1 axis and downstream effectors such as myosin IIa. This ODE-based signaling model is informed by experimental data for specific molecular species, such as the kinetics of CD47-SIRP*α* binding (15). However, purely ODE-based formulations cannot directly capture spatial diffusion of molecules or the physical deformation and contact between a macrophage and a RBC. To address these limitations, we integrated a dissipative particle dynamics (DPD) framework to model the macrophage microenvironment and the mesoscopic interaction between the RBC and macrophage. DPD is a particle-based simulation technique particularly well-suited for soft biological matter (14, 16). In our context, membranes of the macrophage and RBC are represented as collections of interacting particles, while signaling molecules are explicitly simulated as diffusing particles in the surrounding fluid. This captures spatial and stochastic aspects of molecular transport and cell contact that influence downstream signaling. We validated the DPD component by confirming that simulated molecules undergo random-walk trajectories and that the measured mean squared displacement increases linearly with time, yielding diffusion coefficients consistent with experimentally reported values for comparable biomolecules (17, 18).

While the multiscale ODE–DPD framework provides mechanistic descriptions of membrane interactions and signaling dynamics, translating these simulations into quantitative predictions requires reliable estimation of kinetic parameters. In such models, parameter estimation is often hindered by both structural and practical nonidentifiability. Structural nonidentifiability refers to situations in which parameters cannot be uniquely recovered even under idealized, noise-free conditions given the model equations (19, 20). Practical nonidentifiability, in contrast, arises from finite and noisy data, limited observables, or unsuitable experimental design, leading to parameter correlations or flat likelihood regions despite theoretical recoverability (21). In nonlinear dynamical systems such as the signaling model considered here, likelihood surfaces may exhibit extended ridges, parameter compensation, or strongly correlated directions (22, 23). Local sensitivity-based measures such as the Fisher Information Matrix (FIM), and bootstrap or Monte Carlo variance estimates, can both miss near-nonidentifiable manifolds when several parameter combinations explain the data equally well (23, 24).

To obtain a robust assessment, we combine sensitivity-based FIM analysis with profile likelihood, which systematically explores the likelihood surface through repeated reoptimization and enables the identification of finite confidence intervals and functional parameter dependencies (23, 25). These identifiability analyses directly inform parameter selection, search constraints, and the design of the subsequent physics-informed inverse modeling strategy.

Building on this identifiability-aware framework, we employ physics-informed neural networks to infer unknown kinetic parameters while enforcing consistency with the governing ODE system. Physics-Informed Neural Networks (PINNs) enable simultaneous inference of system states and parameters by embedding the differential equations directly into the training objective through automatic differentiation, which is particularly advantageous under partial observation and noisy measurement regimes. Unlike conventional optimization approaches, this formulation does not require repeated numerical integration of the ODE system during optimization, since the solution trajectories are directly represented by the network while satisfying the governing equations through the physics-informed loss. Physics-informed learning has emerged as a powerful paradigm for solving forward and inverse problems in nonlinear dynamical systems by embedding governing equations directly into the loss function (26, 27). In biomedical and systems biology contexts, physics-informed learning has been successfully applied to gray-box identification (28, 29), inverse problems in systems biology and pharmacology (30–32), and mechanistic inference under sparse data conditions (33). We further investigate how neural architecture influences inverse problem robustness by comparing standard PINNs with PIKANs (34). Recent work suggests that architectures based on Kolmogorov–Arnold representations can improve expressivity and stability in nonlinear inverse problems (35–37). In addition to architectural design, we also examine implementation choices that affect inverse-problem stability, including collocation-point selection strategies, with additional experiments provided in the Supplementary material. Although classical optimization methods, such as nonlinear least-squares optimization, particle swarm optimization, differential evolution, and simulated annealing, are well-established approaches for parameter estimation in ODE systems, our motivation for employing PINNs extends beyond parameter estimation alone. In the present multiscale framework, PINNs provide a unified inverse modeling framework that simultaneously estimates unknown kinetic parameters, reconstructs hidden signaling states, enforces the governing ODE system through collocation points, and naturally integrates heterogeneous information sources, including sparse experimental observations, DPD-derived intermediate observables, and initial conditions, within a single optimization problem. Furthermore, the physics-informed formulation naturally accommodates sparse, noisy, and irregularly sampled observations while allowing additional biological constraints or time-varying parameters to be incorporated through the loss function without modifying the overall computational framework.

A novel aspect of our approach is the tight coupling between the DPD simulation and parameter inference in the ODE-based signaling model. Within this framework, PINNs estimate unknown model parameters, such as rate constants and binding affinities, by fitting the ODE system to partially observed trajectories while enforcing consistency with the governing dynamics. In the present study, this inverse analysis is primarily validated using synthetic observations together with DPD-derived trajectories and selected literature-based benchmarks. The PINNs framework learns the time evolution of system states (e.g. concentrations of signaling molecules) such that it not only matches available experimental measurements but also satisfies the ODE constraints (26, 27). This approach is particularly suitable in this context because experimental data on RBC phagocytosis signaling are sparse, often limited to a few time points from assays or microfluidic “spleen-on-a-chip” experiments (8, 38). The DPD simulations further enhance parameter inference by providing spatial–temporal constraints grounded in first-principles physics (39). For example, the DPD model can resolve CD47-SIRP*α* complex formation as a function of cell–cell distance and time, which can then inform ODE model initial conditions or serve as validation inputs (40, 41). Specifically, the DPD simulation tracks receptor occupancy at the macrophage–RBC interface over time, recorded as the fraction of bound receptors; this occupancy trajectory defines the activation state $X_{3}(t)$ of the ODE signaling model, consistent with the definition given in the Supplementary material. The resulting $X_{3}(t)$ time series is then supplied to the PINN as an additional set of time-resolved observations alongside the experimental data, entering the composite loss function as an additional observed-state term whose contribution is controlled by its associated loss weight rather than by the number of simulated samples.

Using this hybrid modeling approach, we investigated how disease-associated perturbations in RBC surface signaling can influence macrophage phagocytosis. Rather than constructing patient-specific disease models, we considered representative perturbations motivated by SCD and GD and represented both through a shared attenuation of the CD47-SIRP*α* signaling complex $X_{3}$. This shared representation is an explicit modeling choice; independently resolving the distinct upstream perturbations of each disease remains a direction for future work. In this setting, the coupled DPD-ODE framework links experimentally motivated changes such as CD47 downregulation in SCD (42) and reduced membrane CD47 expression (43) together with altered RBC surface properties in GD (10, 11) to reduced inhibitory signaling and altered downstream SHP1-mediated responses. These studies show how DPD-derived intermediate observables can be incorporated into predictive signaling dynamics, while the inverse analyses demonstrate that inclusion of $X_{3}$ substantially improves trajectory prediction and parameter recovery (8).

In summary, we developed an integrated multiscale framework coupling DPD simulations of mesoscale membrane dynamics (16) with a mechanistic ODE model of the CD47-SIRP*α* pathway, and infer its unknown kinetic parameters under sparse observations using an identifiability-aware PINNs approach (27), comparing standard PINNs with PIKANs. Incorporating the DPD-derived receptor activation trajectory improves parameter recovery and establishes a computational basis for exploring therapeutic modulation of the CD47-SIRP*α*-SHP1 axis (44). An overview of the integrated modeling approach and representative results is shown in Fig. 1.

**Figure 1:**
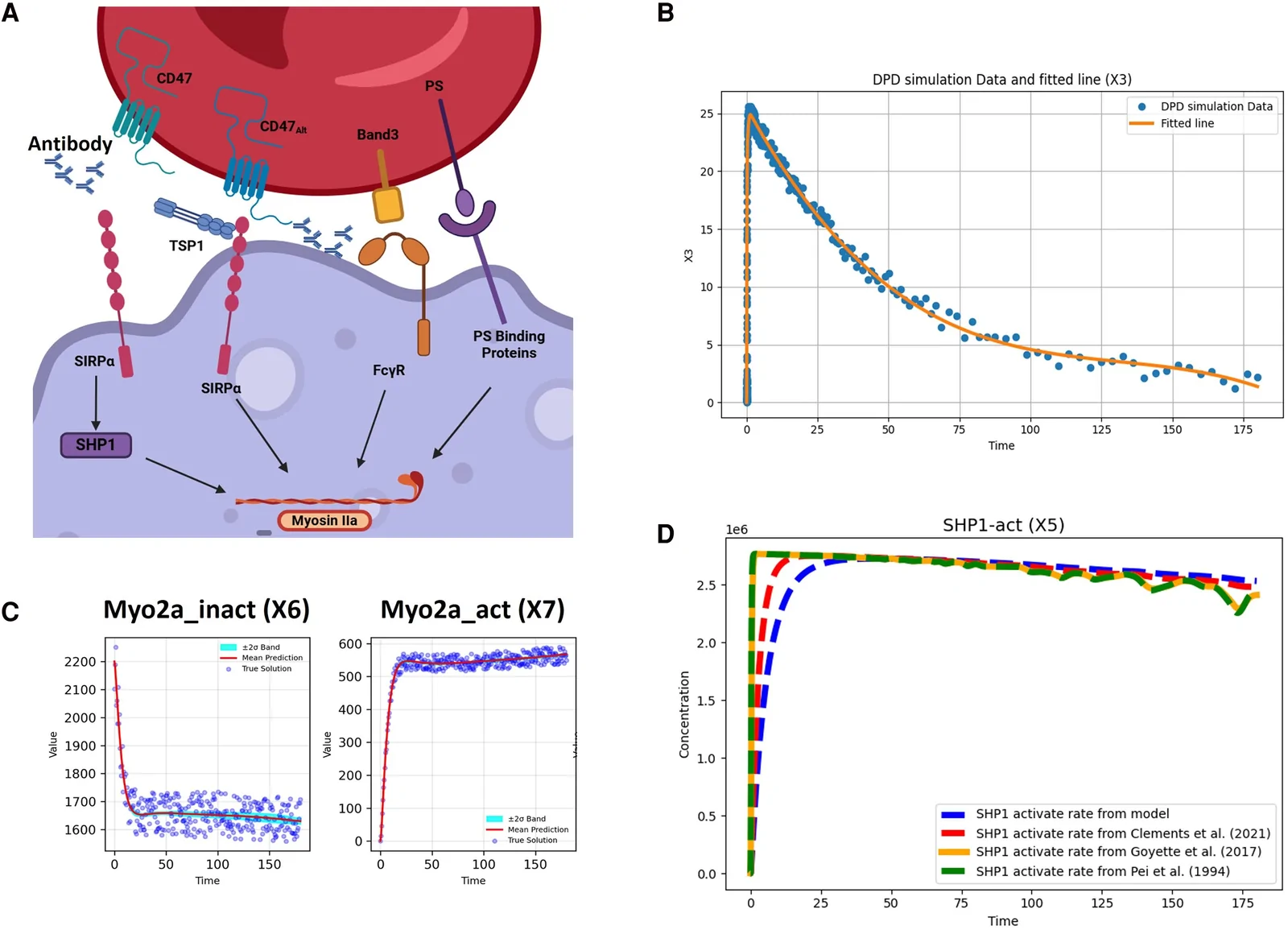
Integrated modeling and data analysis of RBC–macrophage signaling and SHP1 dynamics. A) Schematic illustration of the recognition and signaling pathways regulating RBC clearance. Interactions between CD47-SIRP*α*, phosphatidylserine (PS)-binding proteins, Fc*γ*R, and TSP1 mediate phagocytosis signaling. Downstream, SHP1 activation and myosin IIa regulation contribute to engulfment. B) Dissipative particle dynamics simulation data for binding dynamics (X3) compared with fitted line. C) Physics-informed neural network inference of Myosin IIa dynamics. Left: inactivated Myo2a (Myo2a_inact); Right: activated Myo2a (Myo2a_act). Mean prediction (red) with ±2σ uncertainty band (cyan) is compared with noisy synthetic data (blue dots). D) Comparison of SHP1 activation (X5) kinetics. Model-derived activation rate (blue) is benchmarked against reported values from Clemens et al. (2021, red), Goyette et al. (2017, orange), and Pei et al. (1994, green) (50–52).

## Materials and methods

The framework combines a signaling ODE model, DPD simulations, identifiability analysis, and physics-informed neural networks; each component is described in turn below. To represent the biomechanics of blood cells and membrane interactions we employ the DPD method, widely used for mesoscopic simulations of blood cells and soft matter (8, 14, 45–49); its formulation and parameter values are given in the Supplementary material.

### Signaling ODE model

To describe intracellular signaling events governing erythrophagocytosis, we adopted and extended the systems biology framework introduced in (38), which links receptor-level interactions with downstream cytoskeletal regulation. The model incorporates both inhibitory and prophagocytic signaling pathways involved in macrophage-mediated clearance of RBCs, including the CD47–SIRP*α* inhibitory pathway, SHP1 activation, Fc receptor signaling, phosphatidylserine receptor engagement, and myosin IIa dynamics. The signaling network is formulated as a system of 15 coupled nonlinear ordinary differential equations describing the temporal evolution of receptor complexes and intracellular signaling states. For clarity, the governing equations are summarized in Table S1. In the signaling model, $X_{3}$ represents the activated CD47–SIRP*α* receptor complex that mediates inhibitory signaling. In the DPD simulations, receptor occupancy is used as a mechanistic proxy for this activation state, under the assumption that the fraction of CD47–SIRP*α* binding events correlates with the activation level of the inhibitory signaling pathway. State variables $X_{1}$–$X_{15}$ represent receptor states and intracellular signaling intermediates summarized in Table S3, while parameter definitions and nominal values are provided in Table S2. A detailed list of DPD equations and parameters—including interaction potentials, diffusion time scales, particle densities, and receptor placement methods—is provided in the Supplementary material.

### Identifiability analysis

We first established which parameters are recoverable from the available observables. Structural identifiability was assessed symbolically after reformulating the governing equations to depend rationally on the unknown parameters (19), and practical identifiability under finite, noisy data was assessed with Fisher Information Matrix diagnostics together with profile likelihood (23, 25). Both procedures, including the noise levels, the treatment of $K_{22}$ and the classification criteria, are described in the Supplementary material; the resulting classifications are reported in the Results.

### Physics-Informed Networks (PINs)

To estimate unknown kinetic parameters while enforcing mechanistic consistency with the governing signaling dynamics, we employed Physics-Informed Networks. In this framework, the state trajectories are approximated by a trainable function $\hat{X}(t;\theta )$, and the ODE system is embedded directly into the training objective through automatic differentiation.

As illustrated in Figure 2, the network receives time *t* as input and outputs approximations of the 15 signaling states. Temporal derivatives required for enforcing the ODE constraints are computed via automatic differentiation, allowing evaluation of the residual of the governing system at selected collocation points. The effect of collocation-point selection (fixed grids versus adaptive resampling) is examined in the Supplementary material.

**Figure 2:**
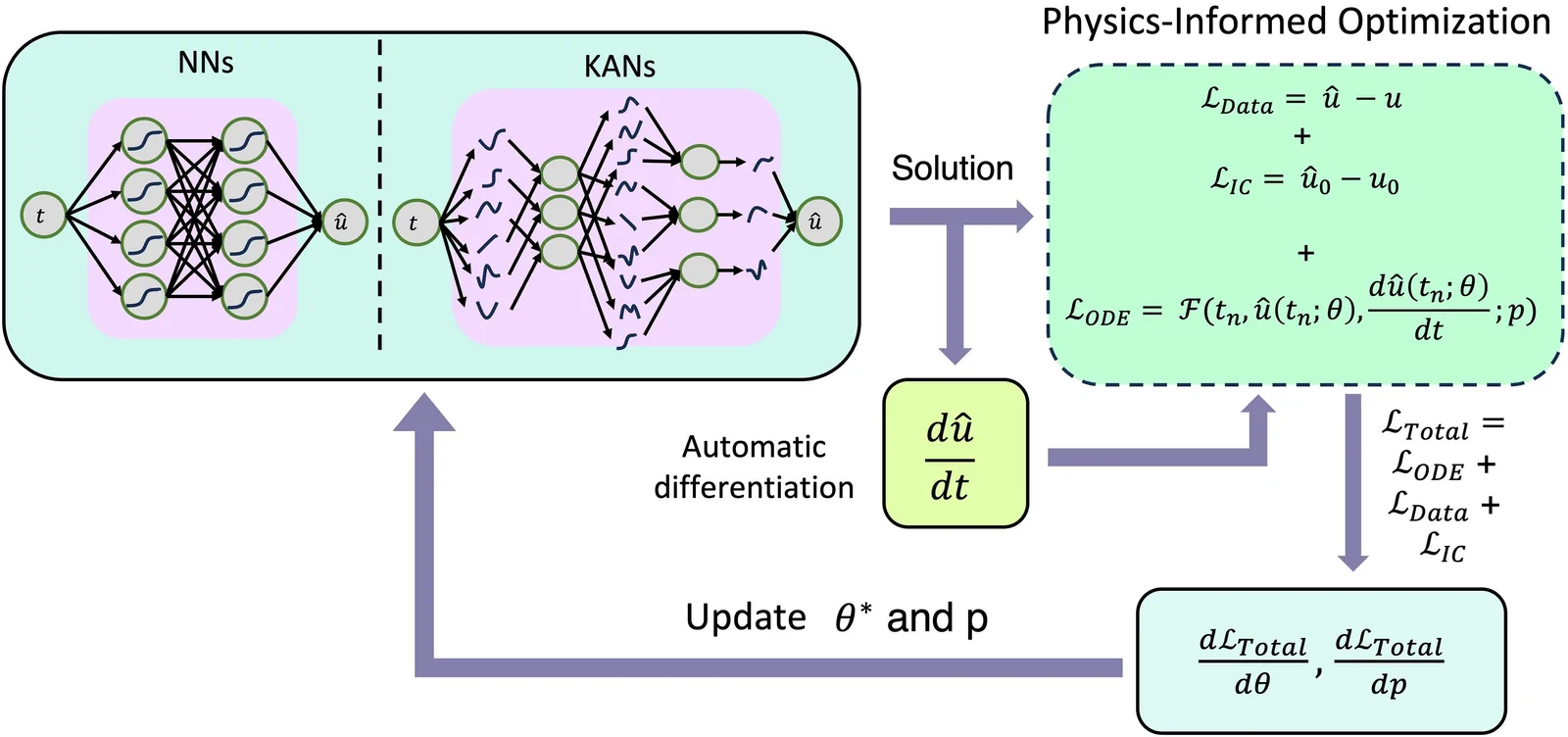
Schematic of the physics-informed networks (PINs) training framework. The network block represents the choice of network architecture used to approximate the system dynamics. Depending on the implementation, this block can employ either neural networks (NNs) or Kolmogorov–Arnold networks (KANs). The selected architecture is then trained within the physics-informed learning framework, where data-driven loss terms are combined with governing equation constraints to guide the optimization. *θ* denotes the trainable parameters of the network, and *p* represents the unknown parameters of the governing mathematical model.

The total loss function is defined as


(1) $$L=\lambda_{\mathrm{data}}L_{\mathrm{data}}+\lambda_{\mathrm{IC}}L_{\mathrm{IC}}+\sum_{k=1}^{N_{\mathrm{eq}}}\beta_{k}L_{\mathrm{ODE}}^{\left(k\right)},$$

where $L_{\mathrm{data}}$ penalizes the mismatch between model predictions and measured observations, $L_{\mathrm{IC}}$ enforces consistency with the initial conditions listed in Table S3, and $L_{\mathrm{ODE}}^{\left(k\right)}$ enforces the residual of the *k*-th governing ODE evaluated at the collocation points. The data and physics terms are given by


(2) $$\begin{array}{rl} L_{\mathrm{data}} & =\sum_{i\in O}\frac{1}{N_{i}}\sum_{n=1}^{N_{i}}{\left|{\hat{X}}_{i}(t_{n};\theta )-X_{i}^{\mathrm{obs}}(t_{n})\right|}^{2},\\ L_{\mathrm{ODE}}^{\left(k\right)} & =\frac{1}{N_{c}}\sum_{\;j=1}^{N_{c}}{\left|R_{k}(t_{j};\theta )\right|}^{2}, \end{array}$$

where O is the set of observed states, $N_{i}$ the number of observations available for state *i*, $N_{c}$ the number of collocation points, and $R_{k}$ the residual of the *k*-th equation; each state is scaled by its maximum value so that the individual terms are dimensionless and comparable in magnitude. The weights are fixed hyperparameters, set here to $\lambda_{\mathrm{data}}=0.1$, $\lambda_{\mathrm{IC}}=1$ and $\beta_{k}=1$ for all $N_{\mathrm{eq}}=15$ equations. The DPD-derived trajectory $X_{3}(t)$ enters $L_{\mathrm{data}}$ on the same footing as the experimental observations. Because each observed state contributes through an average over its own samples rather than a sum, the influence of the DPD-derived observable on the inferred parameters is set by its loss weight and not by the number of simulated samples, which could otherwise be increased arbitrarily at no experimental cost. This composite loss formulation naturally integrates heterogeneous sources of information, including experimental observations, DPD-derived trajectories, governing ODE constraints, and initial conditions, while additional biological constraints, conservation laws, or time-varying parameters can be incorporated through additional loss terms without modifying the overall optimization framework. Within this framework, we compare two representation models for $\hat{X}(t;\theta )$: (i) a standard multilayer perceptron (MLP), and (ii) a Chebyshev-based Kolmogorov–Arnold Network with nested tanh nonlinearities (tanh-cPIKANs) (36, 53), which outperforms the standard Chebyshev-based architecture on ODE-based inverse problems. Additional architectural details are provided in the Supplementary material.

### Inverse problem setup

Following the structural identifiability analysis, we formulated the inverse problem by restricting attention to a subset of parameters deemed estimable under the available measurement configuration. We assume that nine of the fifteen state variables are experimentally measurable. In addition, the time-resolved trajectory of $X_{3}$ is obtained from the DPD simulations. Consequently, observational data are available for the following ten state variables:


$$X_{1},X_{3},X_{4},X_{5},X_{8},X_{9},X_{10},X_{11},X_{14},X_{15}.$$

The parameters selected for estimation are


$$K_{8},K_{9},K_{11},K_{14},K_{15},K_{16},K_{17},K_{23}.$$

Because complete experimental measurements are not available for all observable states, synthetic data were generated by solving the forward problem, which provides synthetic observations for the measurable states together with ground-truth trajectories for the unobserved ones and so enables controlled validation of parameter recovery that sparse experimental datasets cannot support. The DPD-derived trajectory for $X_{3}$ was incorporated into the governing equations, and the full coupled ODE system was integrated forward in time to produce dynamically consistent synthetic data for the remaining observable states. The resulting solution trajectories are shown in Figure 3A.

**Figure 3:**
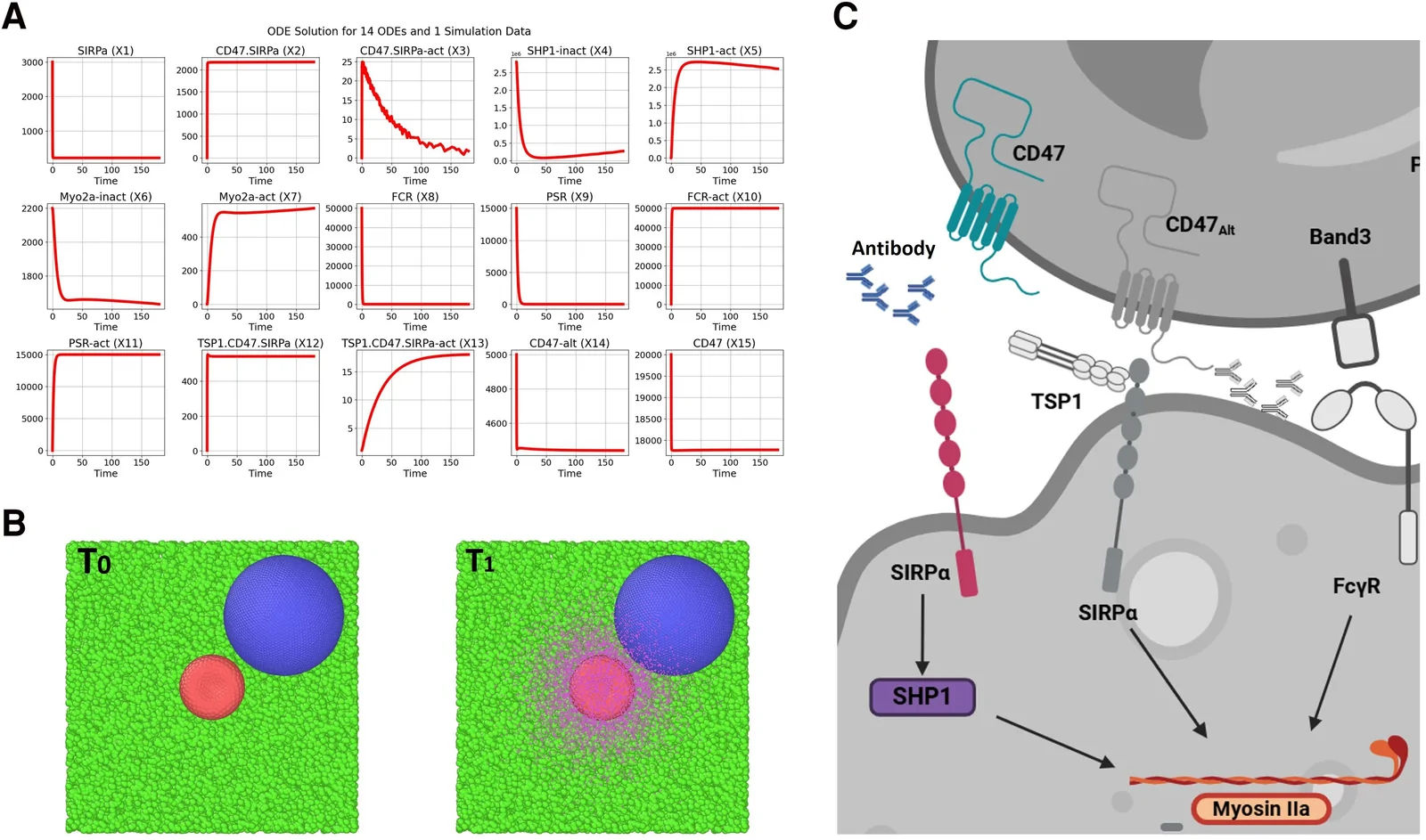
Integrated ODE-based signaling dynamics, particle simulations, and mechanistic signaling pathways. A) Time-course solutions of a 15-variable ODE system modeling the dynamics of RBC-macrophage signaling. X3 (CD47-SIRP*α* complex) includes interpolated experimental data for parameter fitting. B) DPD simulation snapshots showing RBC (red) and macrophage (blue) membrane interactions within a green fluid particle environment at time points t=0 s and t=1 s. Diffusing signaling molecules and particle-level interactions are visualized. C) Mechanistic diagram of the CD47-SIRP*α*-SHP1 signaling axis, with inactive molecules grayed out to indicate early time points prior to engagement.

Nominal values, search ranges and initial conditions are given in Tables S2 and S3.

## Results

### Estimation and validation of diffusion coefficients

In our multiscale framework, the diffusion and binding of antibody particles are simulated specifically during phagocytosis. This localized process is initiated at T=0, corresponding to the onset of sensing between the macrophage pseudopodia and the red blood cell (RBC) membrane. Figures 3B and 3C illustrate the receptor–ligand dynamics and representative RBC–macrophage configurations obtained from the DPD simulations. Rather than simulating the computationally expensive bulk diffusion of antibodies in the entire fluid domain, we initialize the therapeutic particles at the RBC boundary. This specific initial condition represents the localized high effective concentration of opsonizing antibodies confined within the narrow space immediately following T=0. As these particles dynamically diffuse and bind to the macrophage surface receptors in the DPD simulation, they progressively occupy the available binding sites. This physical occupancy induces steric hindrance, preventing native CD47-SIRP*α* ligation. Consequently, the DPD-derived spatial receptor occupancy serves as a spatiotemporal boundary condition that mechanistically dictates the temporal evolution of the inhibitory signaling complex, $X_{3}(t)$, in our ordinary differential equation (ODE) system. By employing this multiscale coupling, our model bridges the microsecond-scale spatial constraints with the minute-scale intracellular signaling dynamics without relying on well-mixed assumptions. This approach inherently captures the mechanosignal coupling where cellular physical contact precedes and governs localized biochemical signaling (54).

To characterize the diffusive behavior captured by the DPD simulations, we analyzed particle trajectories, radial distributions, and mean squared displacement (MSD) statistics, as illustrated in Figure S1. Representative particle trajectories in Figure S1A exhibit typical 3D random walk behavior. As diffusion progresses, particles spread radially from their initial location, leading to the isotropic density distribution shown in Fig. S1B. The radial symmetry of this distribution confirms that the simulated transport is consistent with Brownian diffusion. A quantitative characterization of diffusion is obtained from the MSD curves shown in Fig. S1C, which correspond to three representative particle sizes (10, 12, and 15 nm).

### Validation of antibody dynamics

The interaction between CD47 on target cells and SIRP*α* on macrophages provides a critical inhibitory “don’t eat me” signal that suppresses phagocytosis (4, 44). Engagement of CD47 with SIRP*α* activates inhibitory signaling in macrophages, preventing the clearance of self cells (55). In our multiscale signaling model, this inhibitory pathway is represented by the activation state of the CD47–SIRP*α* complex, denoted as $X_{3}$. Direct observation of antibody diffusion and receptor binding at cell membranes remains experimentally challenging due to nanometer spatial and microsecond temporal scales (56). To investigate these processes and supply critical inputs to our ODE signaling model, we employed DPD simulations. To demonstrate the versatility of our framework, we investigate the disrupted phagocytic signaling observed in both GD and SCD. While GD and SCD have fundamentally different pathophysiological origins, both conditions are characterized by a compromised CD47–SIRP*α* inhibitory axis. In GD, experimental studies reveal a general decrease in membrane CD47 expression on RBCs by 1% to 26% compared to normal cells (43). GD is also associated with elevated autoantibodies (IgG1, IgG2, IgG3); however, as Pandey et al. showed, these are primarily directed against the accumulated substrate glucosylceramide rather than serving as classical anti-RBC opsonins (57). Dupuis et al. (11) further reported that enhanced erythrophagocytosis of GD RBCs was largely independent of opsonization and CD47–SIRP*α* changes, correlating instead with reduced deformability and altered morphology. These findings suggest that CD47–SIRP*α* attenuation may act alongside, rather than as the sole driver of, enhanced GD-RBC clearance. Conversely, in SCD, the disrupted signaling is primarily driven by an inherent reduction of CD47 expression on the RBC surface, while macrophage SIRP*α* expression remains normal (42).

Our primary objective is to establish a unified computational framework that effectively connects DPD-derived observables to the ODE signaling model. To demonstrate the biological utility of this approach, we utilized DPD simulations to capture a critical functional consequence common to both GD and SCD: the attenuation of the $X_{3}$ signaling complex. We note that both diseases are represented here through a single shared $X_{3}$ perturbation rather than independently parameterized disease-specific inputs; the shared attenuation is therefore a modeling simplification, not an emergent prediction. Figure S2 summarizes these results across temporal, spatial, and signaling dimensions. By utilizing a representative DPD simulation to model the lowered $X_{3}$ activation, we demonstrate how our coupled DPD-ODE framework can successfully incorporate experimental disease markers—whether originating from CD47 downregulation in SCD or from the reduced CD47 expression and altered surface properties associated with GD—into predictive signaling dynamics for macrophage phagocytosis.

### Validation of downstream signaling dynamics through SHP1 activation and pathway crosstalk

To further validate our multiscale signaling framework, we examined the activation dynamics of SHP1, a key downstream effector of CD47–SIRP*α* interactions, and compared simulation outputs with experimental measurements. Figure 4 summarizes these results through both quantitative ODE solutions and a schematic overview of the signaling cascade.

**Figure 4:**
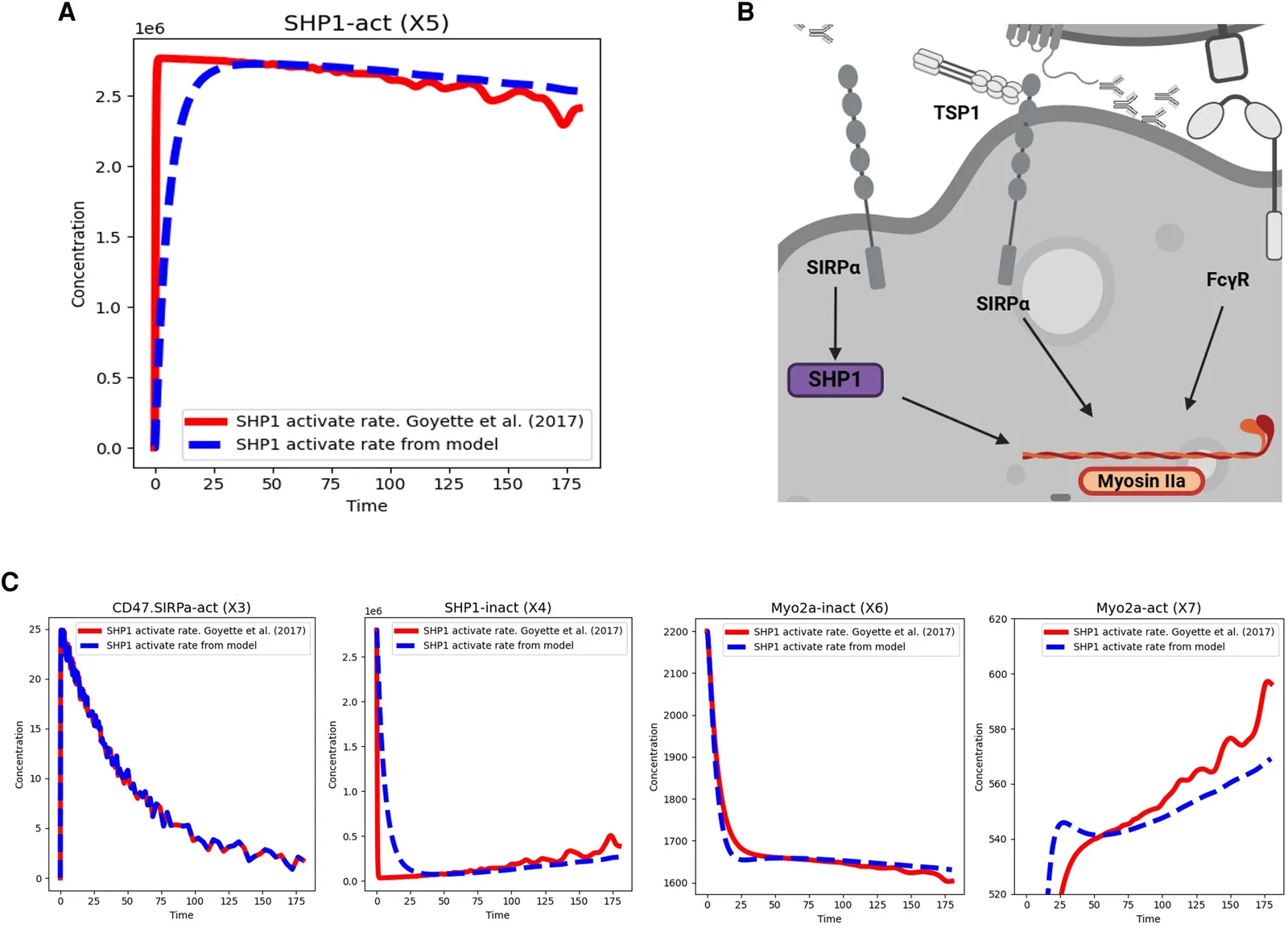
Effect of SHP1 activation rate on RBC clearance signaling dynamics. A) Comparison of SHP1 activation (X5) based on the model (blue) and the experimentally reported rate from Goyette et al. (2017, red). B) Schematic diagram of the SHP1-mediated signaling network downstream of SIRP*α*, highlighting the effect of SHP1 activation rate on Myosin IIa activation. C) Comparison of ODE simulation results for key variables (X3: CD47-SIRP*α* complex activation, X4: SHP1 inactivation, X6: Myo2a inactivation, X7: Myo2a activation) for different SHP1 activation rates. In each case, the model-derived solution (blue) is benchmarked against the rate from Goyette et al. (2017, red), showing how SHP1 kinetics influence molecular responses.

Figure 4A shows the time evolution of activated SHP1 (X5) in response to receptor engagement. The model predicts a rapid increase in SHP1 activation, reaching a steady state within the first 50 time units. This trajectory closely follows experimental measurements reported by Goyette et al. (51), with the model reproducing both the sharp onset of activation and the subsequent plateau. The agreement indicates that the estimated SHP1 activation rate in our framework captures the experimentally observed dynamics. The pathway schematic in Fig. 4B highlights how SIRP*α* engagement recruits and activates SHP1, which in turn modulates downstream effectors including Fc*γ*R and Myosin IIa. This cascade forms the inhibitory arm of the phagocytic checkpoint, integrating antibody blockade, receptor engagement, and cytoskeletal remodeling. To probe the robustness of this pathway representation, we compared ODE solutions for multiple downstream species under different SHP1 activation rates. Figure 4C shows CD47-SIRP*α*-act (X3), SHP1-inact (X4), Myo2a-inact (X6), and Myo2a-act (X7). In each case, the red curves represent experimental activation dynamics from Goyette et al. (51), while the blue curves show simulation results. Across all readouts, the model reproduces the qualitative temporal patterns: rapid depletion of inactive pools (X4, X6), concurrent accumulation of active forms (X5, X7), and sustained reduction of receptor–ligand complexes (X3). Quantitative discrepancies at later times likely reflect simplifications in our current parametrization of SHP1 turnover and cytoskeletal feedback, which can be addressed in future iterations by incorporating experimentally measured dephosphorylation rates and actin remodeling constraints. Overall, these results demonstrate that the model captures the essential features of SHP1-mediated inhibitory signaling. The agreement with experimental datasets, combined with the mechanistic pathway representation, validates the use of our ODE framework to study antibody-mediated modulation of SIRP*α* signaling and its downstream cytoskeletal consequences.

### Structural and practical identifiability analysis

Structural identifiability analysis was performed to determine which kinetic parameters can, in principle, be uniquely recovered from ideal noise-free measurements under the chosen observable configuration. The results under three modeling scenarios are summarized in Table S5. Importantly, this reduction step does not bias the inference process; rather, it ensures that only theoretically recoverable parameters are estimated from data. However, structural identifiability alone does not guarantee reliable parameter recovery in practice. Real experimental measurements are finite, noisy, and often incomplete. Even parameters that are structurally identifiable under ideal noise-free conditions may become weakly constrained when estimation is performed with limited data and measurement uncertainty. Therefore, we next assess practical identifiability, which quantifies the extent to which parameters can be estimated with acceptable uncertainty under realistic observational noise.

We evaluated practical identifiability of the selected kinetic parameters under three observational noise levels (1, 5, and 10%). Parameter estimation was performed using nonlinear least-squares optimization, followed by local curvature analysis via the FIM and global assessment through profile likelihood. Across all noise levels, the optimization converged to finite-cost solutions, although deviations from nominal parameter values increased as noise amplitude increased. The FIM condition numbers were on the order of $10^{5}$–$10^{6}$, indicating substantial ill-conditioning and suggesting the presence of poorly constrained parameter combinations. The FIM correlation heatmaps (Fig. 5) show a stable correlation structure across noise levels.

**Figure 5:**
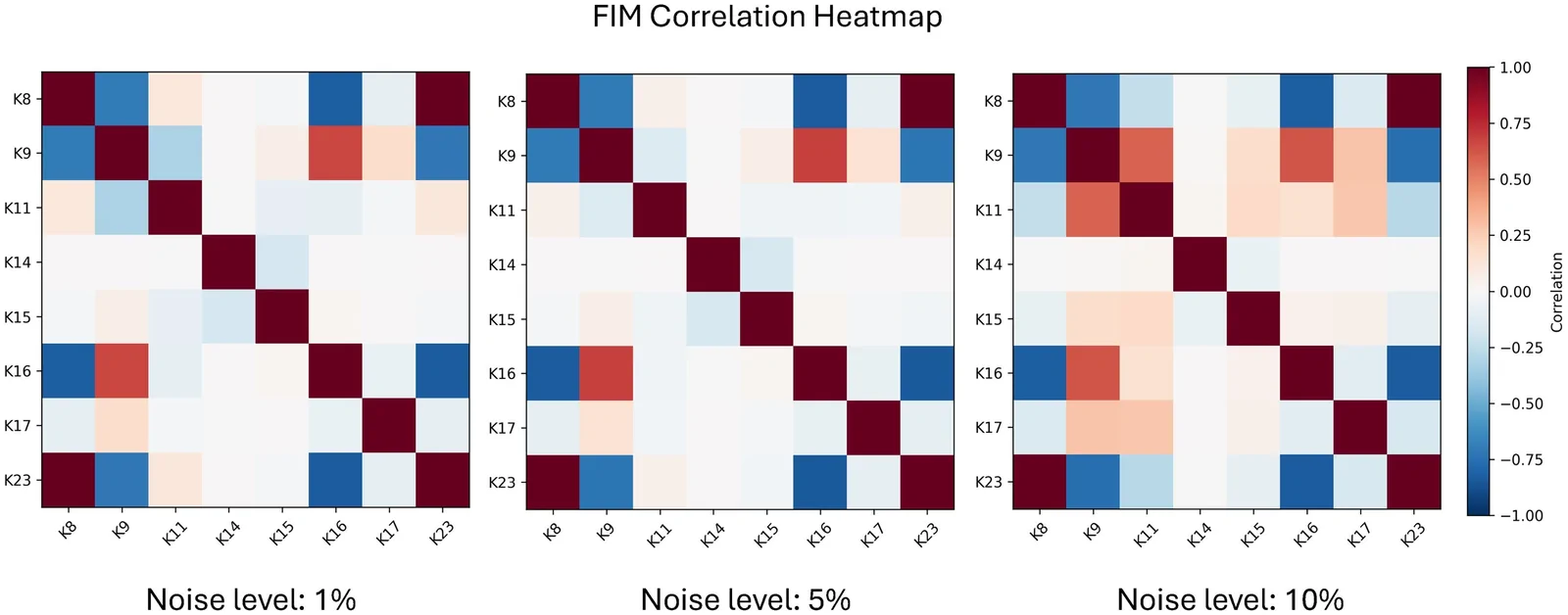
FIM correlation heatmaps for the selected structurally identifiable kinetic parameters under 1%, 5%, and 10% observational noise levels (left to right). Each panel displays the normalized parameter correlation matrix computed at the optimal fit. Strong positive correlation is consistently observed between $K_{8}$ and $K_{23}$ and between $K_{9}$ and $K_{16}$, while $K_{8}$ and $K_{16}$ exhibit pronounced negative correlation. The overall correlation structure remains stable across noise levels, indicating intrinsic parameter coupling in the model rather than noise-induced artifacts.

The agreement between FIM-based curvature diagnostics and profile likelihood confirms the distinction between locally and globally constrained parameters. Table S6 summarizes the fitted parameter values, FIM-based standard deviations, profile likelihood outcomes, and agreement assessment across noise levels. Collectively, these results demonstrate that while several parameters remain practically identifiable under moderate noise, specific kinetic rates, particularly $K_{11}$ and $K_{15}$, are weakly informed by the available observables and are highly sensitive to measurement uncertainty.

### Effect of search space constraints on parameter stability

The practical identifiability analysis revealed substantial ill-conditioning in the reduced parameter set, characterized by large FIM condition numbers ($∼10^{5}$–$10^{6}$), strong parameter correlations (notably between $K_{8}$ and $K_{23}$), and weak curvature directions for $K_{11}$. These findings indicate the presence of compensation effects and flat likelihood directions that can destabilize unconstrained optimization.

To mitigate variance inflation and compensation-induced parameter drift, we introduced search limits for parameters $K_{8}$, $K_{11}$, and $K_{23}$, which showed the strongest instability in the practical identifiability analysis. All remaining parameters were allowed to vary freely within broad admissible ranges.

Table S7 compares parameter estimates obtained with and without search limits. Without constraints, significant bias and dispersion are observed for the red-highlighted parameters. In particular, $K_{8}$ is overestimated (mean $7.16\times 10^{-1}$ vs. true $4.00\times 10^{-1}$) with substantial variability, and $K_{11}$ exhibits nearly threefold overestimation and large standard deviation ($7.02\times 10^{-4}$). Similarly, $K_{23}$ drifts downward with inflated variance.

Imposing search limits substantially improves both accuracy and stability. For $K_{8}$, the mean estimate shifts closer to the true value ($3.83\times 10^{-1}$) and the standard deviation decreases by more than fourfold. The effect is even more pronounced for $K_{11}$, whose standard deviation collapses from $7.02\times 10^{-4}$ to $9.00\times 10^{-6}$, indicating removal of a flat curvature direction. Likewise, the standard deviation of $K_{23}$ decreases by approximately 75%, and its mean estimate aligns closely with the nominal value.

Importantly, parameters that were already well-conditioned (e.g. $K_{9}$, $K_{14}$, $K_{16}$, $K_{17}$) remain stable under both settings, as we expected that the imposed limits do not artificially bias well-identified directions.

The results show that search space restriction, when guided by identifiability diagnostics, acts as a regularization mechanism that suppresses compensation effects and stabilizes inverse inference without compromising mechanistic interpretability.

### Impact of incorporating $X_{3}$ simulation results on prediction and parameter inference

A key question in modeling complex biological signaling systems is whether incorporating additional intermediate-state observables can improve predictive performance. In our system, $X_{3}$ corresponds to the activated CD47-SIRP*α* complex, a central hub in the inhibitory signaling cascade. Biologically, this state represents the intracellularly active form of the CD47–SIRP*α* immune checkpoint, which plays a crucial role in regulating phagocytosis of RBCs by macrophages. Although such intermediate complexes are rarely measured directly in experiments due to technical limitations, they provide mechanistically rich information that could anchor the model and constrain parameter estimation. The simulation results enable controlled validation of identifiability and inference performance, which is not feasible with sparse experimental datasets. We therefore investigated how the inclusion of $X_{3}$ as an observable affects the outcome of PINNs-based parameter inference. To quantify these effects, we designed an uncertainty quantification (UQ) study where each training scenario was repeated 10 times with different random seeds. This procedure captures stochastic variability from initialization and optimization, providing not only average predictions but also distributions of possible outcomes. Such UQ analyses, widely used in physics-informed machine learning frameworks (58, 59), offer a rigorous means of assessing both accuracy and robustness. To further stabilize PINNs training, we employed a smooth *q*-scheduling strategy that gradually increases the weight of the physics-based loss relative to the data loss during optimization; a detailed comparison of different *q*-scheduling strategies is provided in Figure S3 in the Supplementary material.

Figures 6 and 7 summarize the results, obtained using the PINNs framework with 1,000 residual points and 100,000 training epochs. Figure 6 shows predicted trajectories of all model species, with $X_{3}$ highlighted as a representative readout. Without incorporating $X_{3}$ simulation results (Figure 6A), trajectories diverge from the ground truth (blue), but this discrepancy largely reflects parameter misestimation rather than dynamics alone. In particular, parameters such as $K_{8}$, $K_{16}$, and $K_{23}$ exhibit strong bias, which propagates into large steady-state errors and inter-run variance reaching nearly 20%. Including $X_{3}$ simulation results (Figure 6B) restores close agreement in trajectories, with deviations below 10% and markedly reduced variance, reflecting the improved parameter recovery.

**Figure 6:**
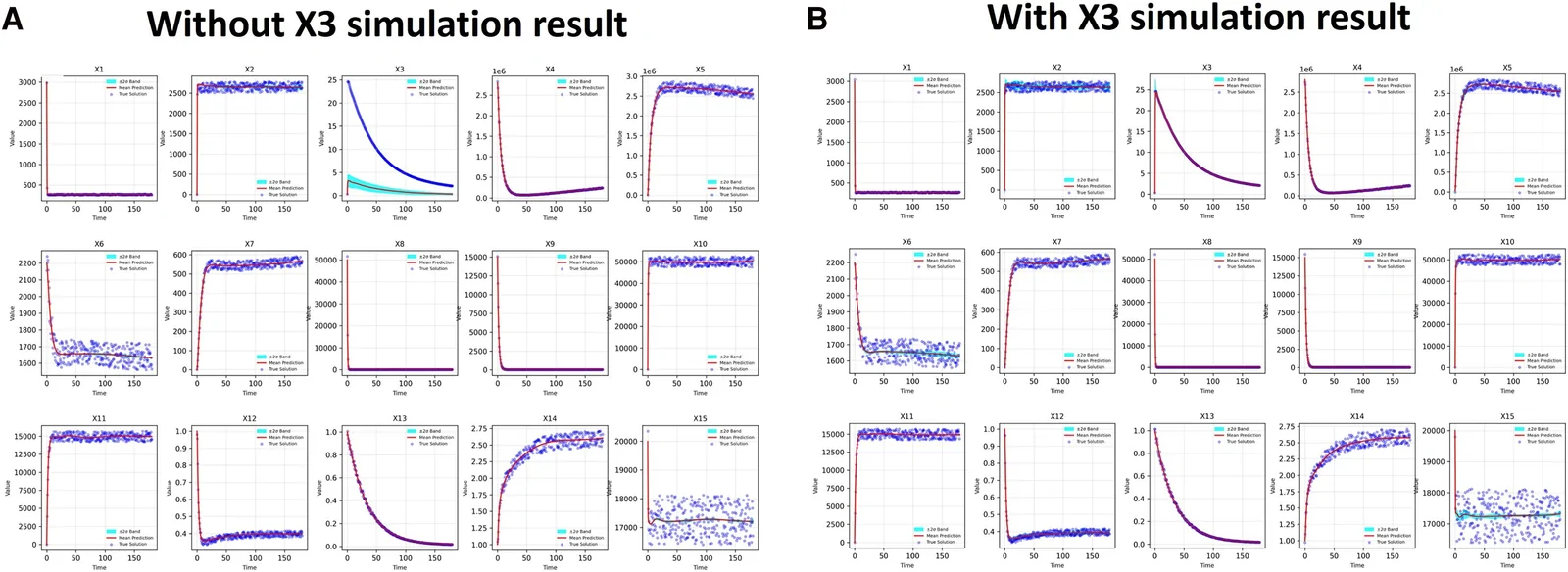
Impact of incorporating $X_{3}$ simulation results on model prediction. A) Model predictions (mean±SD, red) without $X_{3}$ simulation result, compared to ground truth (blue). B) Model predictions with $X_{3}$ simulation result, showing improved accuracy and reduced uncertainty across runs.

**Figure 7:**
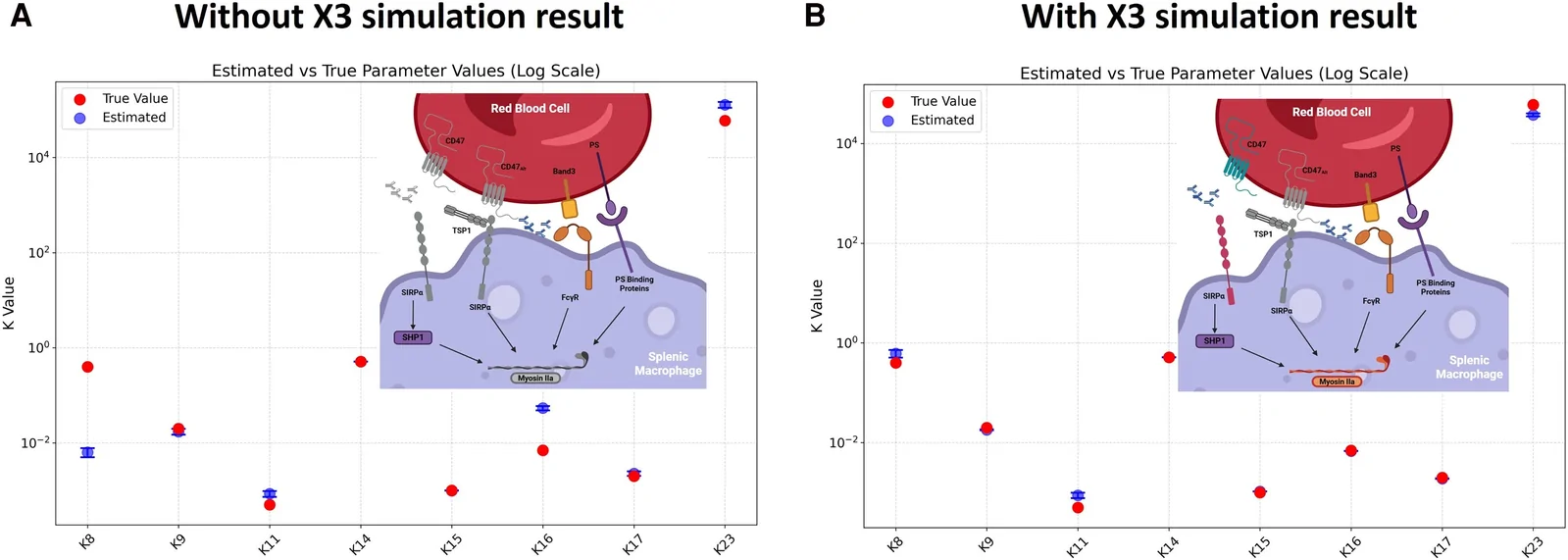
Impact of incorporating $X_{3}$ simulation results on parameter inference. A) Corresponding parameter estimates (blue) with standard deviation bars, plotted against true parameter values (red) on a logarithmic scale without $X_{3}$ simulation result. B) Parameter inference with $X_{3}$ simulation result, showing reduced variance and improved agreement with ground truth, particularly for key parameters ($K_{8}$, $K_{16}$, and $K_{23}$). Insets in (A, B) illustrate the biological context of the CD47-SIRP*α*-SHP1 pathway.

Figure 7 directly compares inferred kinetic parameters against their true values. Without $X_{3}$ simulation results (Fig. 7A), estimates for several parameters—notably $K_{8}$, $K_{16}$, and $K_{23}$—exhibit large bias and variance. For example, $K_{8}$ is underestimated by nearly an order of magnitude, with a standard deviation spanning 1.4 log-units, while $K_{23}$ estimates show variance exceeding 60% relative to the mean. Incorporating $X_{3}$ simulation results (Figure 7B) markedly improves recovery: the bias in $K_{8}$ falls below 15%, variance in $K_{16}$ decreases by 95%, and variance in $K_{23}$ shrinks by over 70%. Overall, parameter estimates cluster tightly around ground truth values, confirming improved identifiability (60) and robustness of the inverse modeling process (27). Thus, the inclusion of even a single mechanistic observable propagates constraints throughout the model, tightening the solution space and stabilizing predictions of unmeasured species. This view makes clear that the trajectory deviations in Fig. 6 are driven by biased parameter estimates.

Taken together, these results demonstrate that $X_{3}$, although representing only a single intermediate species, has an outsized impact on both trajectory prediction and parameter inference. Its inclusion reduces error across the state space, dampens variability between training runs, and dramatically tightens posterior distributions of sensitive kinetic parameters. From a biological perspective, this suggests that targeted measurement of mechanistically central but experimentally challenging observables—particularly those associated with immune checkpoint nodes such as CD47–SIRP*α*—can substantially improve model-based inference. From a computational perspective, the strength of the proposed framework lies in its ability to simultaneously integrate heterogeneous biological information, reconstruct hidden signaling states, enforce governing ODE constraints, and estimate unknown kinetic parameters within a single optimization framework. This is particularly advantageous for multiscale biological systems with sparse and partially observed data. Methodologically, this illustrates how anchoring the network to intermediate signaling states helps recover hidden dynamics and identify parameter sets consistent with experimental reality, and suggests that even a small number of strategically chosen measurements at key mechanistic nodes can substantially increase the reliability of data-driven modeling.

### Robustness to observation noise

To assess the robustness of parameter inference under noisy conditions, we compared the performance of PINNs and Tanh-cPIKANs at different levels of observational noise: 0, 5, and 10%. For both methods, we used 1,000 fixed collocation points uniformly distributed over the time interval t∈[0,180].

To simulate a more realistic scenario, we perturbed synthetic measurement points using noise drawn from a uniform distribution. Specifically, we applied noise according to the following rule:


(3) $$y_{\mathrm{noise}}=y\cdot (1+n\xi ),$$

where *y* is the true model output, *n* is the noise level (e.g. 0.00, 0.05, 0.10), and ξ∼U(−1,1) is a random variable sampled from a uniform distribution. This formulation ensures that the perturbed signals remain non-negative, which is critical when the underlying quantities are biologically constrained to be positive and may approach values close to zero.

Quantitative comparisons are presented in Table S8. At zero noise, the PINNs model generally achieves lower relative errors across most parameters. For instance, PINNs estimates $K_{11}$ and $K_{15}$ with errors of 0.76 and 0.18%, respectively, compared to 12.50% and 5.17% for tanh-cPIKANs. Similarly, PINNs shows lower errors for $K_{9}$, $K_{17}$, and $K_{23}$ in the noise-free case. However, as observational noise increases, the relative performance of the two methods shifts. At 10% noise, tanh-cPIKANs show improved stability in several parameters. For example, the error in estimating $K_{9}$ increases more sharply in PINNs (from 1.86% to 7.00%) than in tanh-cPIKANs (from 2.42% to 3.09%). Additionally, parameters like $K_{14}$ and $K_{16}$ remain within a tight error band (<2.2%) for tanh-cPIKANs across all noise levels, whereas their PINNs counterparts exhibit more variability. It is worth noting that $K_{11}$ remains better estimated by PINNs across all noise levels, with errors staying below 2.5%, while tanh-cPIKANs yield higher errors around 13%. Conversely, for some parameters such as $K_{8}$ and $K_{17}$, the two methods perform comparably or trade off depending on the noise level. Overall, while PINNs tend to outperform in low-noise settings, tanh-cPIKANs exhibit greater robustness and consistency under increasing noise—an important property when dealing with real-world data containing measurement uncertainty.

## Discussion and summary

In this study, we show that incorporating physically derived intermediate observables (X3) fundamentally improves parameter identifiability and predictive accuracy in signaling models.

The integration of dissipative particle dynamics (DPD) simulations with PINNs addresses a fundamental challenge in systems biology: bridging the gap between mesoscale physical interactions and intracellular signaling dynamics. Traditional ordinary differential equation (ODE) models are effective in capturing average biochemical kinetics but often lack the spatial and stochastic resolution required to describe cell–cell interactions at the macrophage–RBC interface. By incorporating DPD-derived trajectories of receptor binding and molecular diffusion, our framework introduces physically grounded constraints into the signaling model, enabling a more realistic representation of how membrane mechanics and molecular transport influence signaling activation. In particular, the DPD-generated trajectory of the CD47-SIRP*α* complex activation state $X_{3}$ provides an intermediate mechanistic observable that anchors the inverse problem and substantially improves parameter inference.

A key mechanistic insight emerging from this study is the central regulatory role of the CD47-SIRP*α*-SHP1 signaling axis in determining macrophage responses to RBCs. The simulations demonstrate that small perturbations in the activation dynamics of SHP1 ($X_{5}$) can propagate through the signaling cascade to significantly influence myosin IIa activation and the formation of the phagocytic cup. Because SHP1 acts as a phosphatase that counterbalances prophagocytic signaling pathways, its activation effectively determines whether macrophages remain in an inhibitory state or proceed toward engulfment. The identifiability results further reinforce this interpretation, and make it quantitative. The parameters governing SHP1 activation and CD47–SIRP*α* signaling—$K_{8}$ (activation rate of CD47-bound SIRP*α*), $K_{16}$ (activation rate of SHP1), and $K_{23}$ (the SHP–SIRP*α* activation coefficient)—are precisely those whose recovery is both most robust and most improved by anchoring the inverse problem to the $X_{3}$ observable. Without $X_{3}$, $K_{8}$ is underestimated by nearly an order of magnitude; incorporating the DPD-derived $X_{3}$ trajectory brings its bias below 15%, reduces the variance in $K_{16}$ by roughly 95%, and shrinks the variance in $K_{23}$ by over 70%, whereas parameters outside this axis change comparatively little. That the single observable carrying information about the CD47–SIRP*α* complex is the one that most sharpens these specific parameters is a direct, quantitative demonstration that the CD47–SIRP*α*–SHP1 axis is the mechanistically central and most identifiable node in the model.

The multiscale framework also provides insight into disease-specific mechanisms underlying pathological RBC clearance. In SCD, the inherent reduction of membrane CD47 expression directly weakens the inhibitory checkpoint signal. Within our multiscale model, this is captured through the attenuated activation of the CD47–SIRP*α* complex ($X_{3}$). This weakened signal manifests as insufficient recruitment of SHP1 to suppress downstream cytoskeletal activation, allowing macrophages to initiate phagocytosis more readily—a result that aligns with the accelerated clearance of SCD RBCs in splenic microenvironments. Conversely, in GD, the compromised checkpoint is modeled as arising from decreased CD47 expression together with increased antibody occupancy at the interface. Concretely, the SCD perturbation is implemented by reducing the initial CD47 surface density in the DPD simulation, which directly lowers the $X_{3}(t)$ trajectory supplied to the ODE model. The GD perturbation additionally raises the density of antibody particles at the RBC boundary. We emphasize that this term is a generic proxy for a reduced-efficacy inhibitory checkpoint under an elevated antibody load, and not a claim that anti-RBC opsonization drives GD clearance: the autoantibodies characteristic of GD are directed against accumulated glucosylceramide rather than the RBC surface (57), and enhanced GD-RBC erythrophagocytosis has been reported to be largely independent of opsonization and of CD47–SIRP*α* changes (11). Both perturbations are therefore represented in the model only through their shared downstream effect—the same reduction in the $X_{3}(t)$ trajectory supplied to the ODE system—which we treat as a modeling simplification rather than a mechanistic assertion about the distinct upstream causes of each disease. Both SCD and GD are represented in our framework through the same attenuation of $X_{3}$ activation. This shared representation is a modeling assumption; demonstrating convergence as an emergent outcome would require independently parameterized disease-specific perturbations, which we identify as an important direction for future work. We also note that the clinical commonalities between SCD and GD—such as ischemia and osteonecrosis—operate at the vascular and tissue level, whereas the present framework addresses clearance at the single-cell macrophage–RBC interface; connecting these scales remains an open challenge. Because GD-specific experimental signaling data suitable for direct model calibration are not yet available, the GD analysis presented here should be understood as a generalizability demonstration of the framework rather than a validated disease-specific prediction; future validation against GD patient-derived signaling measurements would strengthen these conclusions. By explicitly linking these disease-specific extracellular markers to the partial loss of the “don’t eat me” signal, the model provides a mechanistic explanation for how disrupted receptor engagement shifts the intracellular signaling balance toward RBC engulfment. The DPD simulations indicate that these mechanical alterations modify receptor spatial distribution and effective binding affinity, which in turn shifts the signaling balance toward macrophage activation. These findings highlight the importance of considering both biochemical signaling and physical cell properties when investigating erythrophagocytosis. The simulations establish a quantitative framework for analyzing diffusion and localized binding dynamics. The results are consistent with fluorescence microscopy observations, where receptor–ligand complexes organize into microdomains with elevated local densities (61). While these results support the biological relevance of the model, it is important to note that the current framework does not include an explicit cytoskeletal scaffold. Such structures are known to restrict receptor diffusion and organize membrane domains in RBCs and neurons (62–66), and their absence may contribute to quantitative differences between simulation and experiment. Nevertheless, by utilizing a representative DPD simulation to model the lowered $X_{3}$ activation, we demonstrate how our coupled DPD–ODE framework can successfully incorporate experimental disease markers—whether originating from CD47 downregulation in SCD or from the reduced CD47 expression and altered surface properties associated with GD—into predictive signaling dynamics for macrophage phagocytosis.

An important consideration not yet captured in our framework is the direct mechanical contribution to phagocytic clearance. RBC stiffness and deformability are well-established determinants of splenic filtration and macrophage recognition: Franco et al. (10) showed that GD RBCs exhibit abnormal mechanical properties that promote their retention and clearance, and our recent computational studies demonstrated that GD-associated stiffening increases blood viscosity (14) and significantly alters RBC transit through splenic slits (67). More broadly, Sosale et al. (54) established that cell rigidity can override CD47-mediated inhibition, with stiffer targets being engulfed even when CD47 levels are normal. These findings indicate that the CD47–SIRP*α* checkpoint operates not in isolation but in competition with mechanical cues sensed by the macrophage. Incorporating explicit mechanical inputs—such as DPD-resolved membrane stiffness and contact geometry—into the signaling model would enable the framework to capture this interplay and is a natural extension of the current work, building on DPD-based mechanical characterization that has been linked to splenic filtration and hemorheology across a range of RBC morphologies and mechanical phenotypes (68, 69).

Beyond SCD and GD, the proposed multiscale framework provides a generalizable paradigm for understanding how mechanical and biochemical cues jointly regulate RBC clearance across diverse pathological conditions. By decoupling membrane mechanics from intracellular signaling while preserving their functional coupling through receptor-level interactions, the model enables systematic exploration of how distinct disease-specific perturbations act through common immune checkpoint pathways. This generality is particularly relevant for infectious diseases such as malaria, where *Plasmodium*-infected RBCs exhibit both increased membrane rigidity and altered immune signaling. Previous work suggests that parasites can exploit the CD47–SIRP*α* axis to evade macrophage clearance by preferentially infecting young RBCs that already display high CD47 levels, which shields the infected cells from phagocytosis (70). Within our framework, such effects can be interpreted as a shift in both mechanical constraints and effective inhibitory signaling capacity, allowing the model to predict how competing influences—rigidity-induced retention versus signaling-mediated immune evasion—govern clearance outcomes. Similarly, in metabolic disorders such as diabetes, chronic hyperglycemia drives oxidative stress that promotes RBC membrane vesiculation, accompanied by a reduction in CD47 expression (71). These coupled alterations naturally map onto our framework as simultaneous changes in DPD-resolved membrane properties and baseline inhibitory signaling levels. Importantly, this unified representation suggests that seemingly distinct pathological processes may share a common mechanistic pathway: the disruption of spatially regulated CD47–SIRP*α* signaling at the macrophage interface. Taken together, these examples highlight that the predictive capability of the framework rests on targeted modulation of physically interpretable variables rather than on disease-specific reparameterization of the entire model.

From a methodological perspective, the comparison between standard PINNs and PIKANs reveals complementary advantages. While PINNs tend to achieve lower errors under low-noise conditions, PIKANs exhibit greater robustness to observational noise and high-frequency fluctuations arising from particle-based simulations. This robustness likely arises from the localized functional representation enabled by the Kolmogorov-Arnold architecture, which can capture localized nonlinear features more effectively than global multilayer perceptrons.

Overall, by explicitly linking receptor-level interactions, intracellular signaling dynamics, and parameter inference, the model provides a mechanistic explanation for dysregulated RBC clearance in SCD and GD, and establishes a computational platform for exploring therapeutic interventions targeting macrophage immune checkpoints.

Despite the insights provided by the proposed framework, several limitations should be noted. The signaling network is modeled using deterministic ordinary differential equations that assume spatial homogeneity inside the macrophage, which may overlook spatial heterogeneity and stochastic effects in intracellular signaling. Parameter inference is mainly based on synthetic datasets with limited experimental validation, and some parameters were fixed to address structural nonidentifiability, which may influence model predictions under different biological conditions. In addition, the DPD simulations use a coarse-grained representation of membranes and antibodies, which improves computational efficiency but simplifies molecular details such as cytoskeletal structures and receptor clustering. These assumptions were necessary to ensure computational tractability while retaining the dominant mechanisms governing macrophage-RBC signaling.

Future work will focus on improving biological realism and predictive capability by integrating additional experimental data, such as microfluidic spleen-on-a-chip assays or imaging of macrophage-RBC interactions, to better calibrate and validate the model. Extending the framework to include spatially resolved intracellular signaling, for example through reaction–diffusion or agent-based models, could capture spatial heterogeneity during phagocytic cup formation. Further development of physics-informed machine learning approaches, including uncertainty-aware methods, may also enhance parameter inference and predictive reliability. Finally, the framework could be applied to investigate therapeutic strategies targeting immune checkpoint pathways such as CD47-SIRP*α*. Ultimately, by mathematically anchoring intracellular signaling networks to the physical mechanics of the cell membrane, this approach moves us closer to a truly predictive, multiscale understanding of immune regulation.

## Supplementary Material

pgag348_Supplementary_Data

## Data Availability

The data and code underlying this article are publicly available on GitHub at https://github.com/ZhaojieC/Signaling-Biophysical-Framework and archived on Zenodo at https://doi.org/10.5281/zenodo.23090753, and the computational model used to generate all simulations in this study is available in the Supplementary material.

## Preprints

This manuscript was posted on a preprint: https://doi.org/10.64898/2026.04.20.719505.

## References

[1] Hayes BH, Zhu H, Andrechak JC, Dooling LJ, Discher DE. 2023. Titrating CD47 by mismatch CRISPR-interference reveals incomplete repression can eliminate IgG-opsonized tumors but limits induction of antitumor IgG. PNAS Nexus. 2:pgad243. 10.1093/pnasnexus/pgad243 PMC10427748 37593202

[2] Rodriguez PL, et al 2013. Minimal “self” peptides that inhibit phagocytic clearance and enhance delivery of nanoparticles. Science. 339:971–975. 10.1126/science.1229568 PMC3966479 23430657

[3] Tsai RK, Discher DE. 2008. Inhibition of “self” engulfment through deactivation of myosin-II at the phagocytic synapse between human cells. J Cell Biol. 180:989–1003. 10.1083/jcb.200708043 PMC2265407 18332220

[4] Willingham SB, et al 2012. The CD47-signal regulatory protein alpha (SIRPa) interaction is a therapeutic target for human solid tumors. Proc Natl Acad Sci U S A. 109:6662–6667. 10.1073/pnas.1121623109 PMC3340046 22451913

[5] Barclay AN . 2009. Signal regulatory protein alpha (SIRP*α*)/CD47 interaction and function. Curr Opin Immunol. 21:47–52. 10.1016/j.coi.2009.01.008 PMC3128989 19223164

[6] Oldenborg P-A, et al 2000. Role of CD47 as a marker of self on red blood cells. Science. 288:2051–2054. 10.1126/science.288.5473.2051 10856220

[7] Feng M, et al 2019. Phagocytosis checkpoints as new targets for cancer immunotherapy. Nat Rev Cancer. 19:568–586. 10.1038/s41568-019-0183-z PMC7002027 31462760

[8] Ma S, et al 2023. Computational investigation of flow dynamics and mechanical retention of age-associated red blood cells in the spleen. Phys Rev Fluids. 8:063103.

[9] Khandelwal S, Van Rooijen N, Saxena RK. 2007. Reduced expression of CD47 during murine red blood cell (RBC) senescence and its role in RBC clearance from the circulation. Transfusion. 47:1725–1732. 10.1111/j.1537-2995.2007.01348.x 17725740

[10] Franco M, et al 2013. Abnormal properties of red blood cells suggest a role in the pathophysiology of Gaucher disease. Blood. 121:546–555. 10.1182/blood-2012-07-442467 23212518

[11] Dupuis L, et al 2022. Phagocytosis of erythrocytes from Gaucher patients induces phenotypic modifications in macrophages, driving them toward Gaucher cells. Int J Mol Sci. 23:7640. 10.3390/ijms23147640 PMC9319206 35886988

[12] Adar T, et al 2008. Increased red blood cell aggregation in patients with Gaucher disease is non-inflammatory. Clin Hemorheol Microcirc. 40:113–118. 19029636

[13] Adar T, et al 2006. Aggregation of red blood cells in patients with Gaucher disease. Br J Haematol. 134:432–437. 10.1111/j.1365-2141.2006.06199.x 16827817

[14] Chai Z, et al 2025. In silico biophysics and rheology of blood and red blood cells in Gaucher Disease. PLoS Comput Biol. 21:e1012705. 10.1371/journal.pcbi.1012705 PMC12435781 40929301

[15] Oldenborg P-A, Gresham HD, Lindberg FP. 2001. CD47-signal regulatory protein *α* (SIRP*α*) regulates Fc*γ* and complement receptor–mediated phagocytosis. J Exp Med. 193:855–862. 10.1084/jem.193.7.855 PMC2193364 11283158

[16] Fedosov DA, Caswell B, Karniadakis GE. 2010. A multiscale red blood cell model with accurate mechanics, rheology, and dynamics. Biophys J. 98:2215–2225. 10.1016/j.bpj.2010.02.002 PMC2872218 20483330

[17] Francis K, Palsson BO. 1997. Effective intercellular communication distances are determined by the relative time constants for cyto/chemokine secretion and diffusion. Proc Natl Acad Sci U S A. 94:12258–12262. 10.1073/pnas.94.23.12258 PMC24899 9356436

[18] Pokrić B, Pučar Z. 1979. The two-cross immunodiffusion technique: diffusion coefficients and precipitating titers of IgG in human serum and rabbit serum antibodies. Anal Biochem. 93:103–114. 107826

[19] Daneker M, Zhang Z, Karniadakis GE, Lu L. Systems biology: identifiability analysis and parameter identification via systems-biology-informed neural networks. In: Computational modeling of signaling networks. Springer; 2023. p. 87–105. 10.1007/978-1-0716-3008-2_4 37074575

[20] Miao H, Xia X, Perelson AS, Wu H. 2011. On identifiability of nonlinear ODE models and applications in viral dynamics. SIAM Rev. 53:3–39. 10.1137/090757009 PMC3140286 21785515

[21] Heinrich M, Arutjunjan R, Timmer J. 2025. On the different flavours of practical identifiability. Curr Opin Syst Biol. 42:100556.

[22] Gutenkunst RN, et al 2007. Universally sloppy parameter sensitivities in systems biology models. PLoS Comput Biol. 3:e189. 10.1371/journal.pcbi.0030189 PMC2000971 17922568

[23] Wieland F-G, Hauber AL, Rosenblatt M, Tönsing C, Timmer J. 2021. On structural and practical identifiability. Curr Opin Syst Biol. 25:60–69.

[24] Hines KE, Middendorf TR, Aldrich RW. 2014. Determination of parameter identifiability in nonlinear biophysical models: a Bayesian approach. J Gen Physiol. 143:401–416. 10.1085/jgp.201311116 PMC3933937 24516188

[25] Raue A, et al 2009. Structural and practical identifiability analysis of partially observed dynamical models by exploiting the profile likelihood. Bioinformatics. 25:1923–1929. 10.1093/bioinformatics/btp358 19505944

[26] Karniadakis GE, et al 2021. Physics-informed machine learning. Nat Rev Phys. 3:422–440.

[27] Raissi M, Perdikaris P, Karniadakis GE. 2019. Physics-informed neural networks: a deep learning framework for solving forward and inverse problems involving nonlinear partial differential equations. J Comput Phys. 378:686–707.

[28] Ahmadi Daryakenari N, Florio MD, Shukla K, Karniadakis GE. 2024. AI-Aristotle: a physics-informed framework for systems biology gray-box identification. PLoS Comput Biol. 20:e1011916. 10.1371/journal.pcbi.1011916 PMC10931529 38470870

[29] Raissi M, Perdikaris P, Ahmadi N, Karniadakis GE. 2024. Physics-informed neural networks and extensions [preprint], arXiv, arXiv:2408.16806, https://arxiv.org/abs/2408.16806.

[30] Ahmadi N, Wang S, Karniadakis G. 2024. Pharmacometrics modeling via physics-informed neural networks: integrating time-variant absorption rates and fractional calculus for enhancing prediction accuracy [preprint], arXiv, arXiv:2412.21076, https://arxiv.org/abs/2412.21076.

[31] Ahmadi Daryakenari N, Wang S, Karniadakis G. 2025. CMINNs: compartment model informed neural networks—unlocking drug dynamics. Comput Biol Med. 184:109392. 10.1016/j.compbiomed.2024.109392 PMC11701831 39608036

[32] Przedborski M, Smalley M, Thiyagarajan S, Goldman A, Kohandel M. 2021. Systems biology informed neural networks (SBINN) predict response and novel combinations for PD-1 checkpoint blockade. Commun Biol. 4:877. 10.1038/s42003-021-02393-7 PMC8282606 34267327

[33] Ahmadi N, Cao Q, Humphrey JD, Karniadakis GE. 2026. Physics-informed machine learning in biomedical science and engineering. Annu Rev Biomed Eng. 28:309–336. 10.1146/annurev-bioeng-110824-124907 PMC13455127 42067517

[34] Shukla K, Toscano JD, Wang Z, Zou Z, Karniadakis GE. 2024. A comprehensive and FAIR comparison between MLP and KAN representations for differential equations and operator networks. Comput Methods Appl Mech Eng. 431:117290.

[35] Daryakenari NA, Karniadakis GE. Leveraging Kolmogorov–Arnold networks to discover time-dependent drug efficacy function. In: American Conference on Pharmacometrics. International Society of Pharmacometrics; 2026. p. S-081.

[36] Ahmadi Daryakenari N, Shukla K, Karniadakis GE. 2026. Representation meets optimization: training PINNs and PIKANs for gray-box discovery in systems pharmacology. Comput Biol Med. 201:111393. 10.1016/j.compbiomed.2025.111393 PMC13387123 41447950

[37] Toscano JD, et al 2025. From PINNs to PIKANs: recent advances in physics-informed machine learning. Mach Learn Comput Sci Eng. 1:15. 10.1007/s44379-025-00015-1 PMC13290273 42344579

[38] Zhang Y, et al 2024. Signaling-biophysical modeling unravels mechanistic control of red blood cell phagocytosis by macrophages in sickle cell disease. PNAS Nexus. 3:pgae031. 10.1093/pnasnexus/pgae031 PMC10833451 38312226

[39] Huang S, He Z, Dirr N, Zimmer J, Reina C. 2025. Statistical-Physics-Informed Neural Networks (Stat-PINNs): a machine learning strategy for coarse-graining dissipative dynamics. J Mech Phys Solids. 194:105908.

[40] Hou R, Ren S, Wang R, Różycki B, Hu J. 2025. Multiscale simulations of membrane adhesion mediated by CD47-SIRP*α* complexes. J Chem Theory Comput. 21:2030–2042. 10.1021/acs.jctc.4c01337 PMC11866742 39960300

[41] Li L, Gui C, Hu J, Różycki B. 2023. Membrane-mediated cooperative interactions of CD47 and SIRP*α*. Membranes (Basel). 13:871. 10.3390/membranes13110871 PMC10673186 37999357

[42] Eldakhakhny B, et al 2021. Evaluation of the role of CD47 in sickle cell disease. J Hematop. 14:31–39.

[43] Bratosin D, et al 2011. A cytometric study of the red blood cells in Gaucher disease reveals their abnormal shape that may be involved in increased erythrophagocytosis. Cytometry B Clin Cytom. 80B:28–37. 10.1002/cyto.b.20539 20568298

[44] Qu T, Li B, Wang Y. 2022. Targeting CD47/SIRP*α* as a therapeutic strategy, where we are and where we are headed. Biomark Res. 10:20. 10.1186/s40364-022-00373-5 PMC9009010 35418166

[45] Du Y, et al 2026. High-throughput biomimetic cycling of red blood cells: elucidating the morpho-mechanical determinants of fatigue and clearance. Lab Chip. 26:551–563. 10.1039/d5lc01022e 41532666

[46] Li G, Ye T, Li X. 2020. Parallel modeling of cell suspension flow in complex micro-networks with inflow/outflow boundary conditions. J Comput Phys. 401:109031.

[47] Li G, Ye T, Wang S, Li X, Haq UR. 2020. Numerical design of a highly efficient microfluidic chip for blood plasma separation. Phys Fluids. 32:031903.

[48] Liu ZL, et al 2021. Computational modeling of biomechanics and biorheology of heated red blood cells. Biophys J. 120:4663–4671. 10.1016/j.bpj.2021.09.038 PMC8595741 34619119

[49] Wang S, Ma L, Guo L, Li X, Zhou T. 2026. Multi-stage uncertainty quantification framework for red blood cell morpho-mechanics. Int J Mech Sci. 313:111352.

[50] Clemens L, et al 2021. Determination of the molecular reach of the protein tyrosine phosphatase SHP-1. Biophys J. 120:2054–2066. 10.1016/j.bpj.2021.03.019 PMC8204385 33781765

[51] Goyette J, et al 2017. Biophysical assay for tethered signaling reactions reveals tether-controlled activity for the phosphatase SHP-1. Sci Adv. 3:e1601692. 10.1126/sciadv.1601692 PMC5365251 28378014

[52] Pei D, Lorenz U, Klingmuller U, Neel BG, Walsh CT. 1994. Intramolecular regulation of protein tyrosine phosphatase SH-PTP1: a new function for Src homology 2 domains. Biochemistry. 33:15483–15493. 10.1021/bi00255a030 7528537

[53] Khodakarami S, Oommen V, Daryakenari NA, Beekenkamp M, Karniadakis GE. 2026. Spectral bias in physics-informed and operator learning: analysis and mitigation guidelines. Comput Methods Appl Mech Eng. 461:119156.

[54] Sosale NG, et al 2015. Cell rigidity and shape override CD47’s “self”-signaling in phagocytosis by hyperactivating myosin-II. Blood. 125:542–552. 10.1182/blood-2014-06-585299 PMC4296014 25411427

[55] Chao MP, et al 2020. Therapeutic targeting of the macrophage immune checkpoint CD47 in myeloid malignancies. Front Oncol. 9:1380. 10.3389/fonc.2019.01380 PMC6990910 32038992

[56] Treanor B, et al 2010. The membrane skeleton controls diffusion dynamics and signaling through the B cell receptor. Immunity. 32:187–199. 10.1016/j.immuni.2009.12.005 PMC2984614 20171124

[57] Pandey MK, et al 2017. Complement drives glucosylceramide accumulation and tissue inflammation in Gaucher disease. Nature. 543:108–112. 10.1038/nature21368 28225753

[58] Lu L, Meng X, Mao Z, Karniadakis GE. 2021. DeepXDE: a deep learning library for solving differential equations. SIAM Rev. 63:208–228.

[59] Yang L, Meng X, Karniadakis GE. 2021. B-PINNs: Bayesian physics-informed neural networks for forward and inverse PDE problems with noisy data. J Comput Phys. 425:109913.

[60] Villaverde AF . 2019. Observability and structural identifiability of nonlinear biological systems. Complexity. 2019:8497093.

[61] Varma R, Mayor S. 1998. GPI-anchored proteins are organized in submicron domains at the cell surface. Nature. 394:798–801. 10.1038/29563 9723621

[62] Chai Z, Gu S, Lykotrafitis G. 2023. Dynamics of the axon plasma membrane skeleton. Soft Matter. 19:2514–2528. 10.1039/d2sm01602h 36939651

[63] Chai Z, Tzingounis AV, Lykotrafitis G. 2022. The periodic axon membrane skeleton leads to Na nanodomains but does not impact action potentials. Biophys J. 121:3334–3344. 10.1016/j.bpj.2022.08.027 PMC9515372 36029000

[64] Li H, Lykotrafitis G. 2014. Erythrocyte membrane model with explicit description of the lipid bilayer and the spectrin network. Biophys J. 107:642–653. 10.1016/j.bpj.2014.06.031 PMC4129483 25099803

[65] Li H, Zhang Y, Ha V, Lykotrafitis G. 2016. Modeling of band-3 protein diffusion in the normal and defective red blood cell membrane. Soft Matter. 12:3643–3653. 10.1039/c4sm02201g 26977476

[66] Zhang Y, Tzingounis AV, Lykotrafitis G. 2019. Modeling of the axon plasma membrane structure and its effects on protein diffusion. PLoS Comput Biol. 15:e1007003. 10.1371/journal.pcbi.1007003 PMC6497228 31048841

[67] Chai Z, de Person M, Buffet PA, Franco M, Karniadakis GE. 2026. Quantifying the biophysical properties of red blood cells in Gaucher disease [preprint], arXiv, arXiv:2608.24021, https://arxiv.org/abs/2608.24021.10.1016/j.bpj.2026.10.02142926315

[68] Chai Z, Zheng J, Li H, Dao M, Karniadakis GE. 2026. Quantifying reticulocyte biomechanics in health and disease [preprint], arXiv, arXiv:2607.21810, https://arxiv.org/abs/2607.21810.

[69] Chai Z, Zheng J, Li H, Dao M, Karniadakis GE. 2026. Quantifying the biophysical properties of stomatocytes in health and disease. Biophys J. In press. 10.1016/j.bpj.2026.10.007 PMC13722006 42870423

[70] Banerjee R, Khandelwal S, Kozakai Y, Sahu B, Kumar S. 2015. CD47 regulates the phagocytic clearance and replication of the *Plasmodium yoelii* malaria parasite. Proc Natl Acad Sci U S A. 112:3062–3067. 10.1073/pnas.1418144112 PMC4364205 25713361

[71] Sukati S, et al 2025. Elevated red blood cell-derived extracellular vesicles under hyperglycemic conditions are associated with CD47 expression and production of intracellular reactive oxygen species. Biomed Rep. 23:129. 10.3892/br.2025.2007 PMC12150669 40502359

[72] Wolak DJ, Pizzo ME, Thorne RG. 2015. Probing the extracellular diffusion of antibodies in brain using in vivo integrative optical imaging and ex vivo fluorescence imaging. J Control Release. 197:78–86. 10.1016/j.jconrel.2014.10.034 PMC4272890 25449807

[73] Hoogerbrugge PJ, Koelman JMVA. 1992. Simulating microscopic hydrodynamic phenomena with dissipative particle dynamics. Europhys Lett. 19:155–160.

[74] Español P, Warren P. 1995. Statistical mechanics of dissipative particle dynamics. Europhys Lett. 30:191–196.

[75] Groot RD, Warren PB. 1997. Dissipative particle dynamics: bridging the gap between atomistic and mesoscopic simulation. J Chem Phys. 107:4423–4435.

[76] Lei H, Caswell B, Karniadakis GE. 2010. Direct construction of mesoscopic models from microscopic simulations. Phys Rev E. 81:026704. 10.1103/PhysRevE.81.026704 PMC3693397 20365672

[77] Wu Z-H, et al 2022. Preclinical characterization of the novel anti-SIRPα antibody BR105 that targets the myeloid immune checkpoint. J Immunother Cancer. 10:e004054. 10.1136/jitc-2021-004054 PMC8905892 35256517

[78] Español P, Warren PB. 2017. Perspective: dissipative particle dynamics. J Chem Phys. 146:150901. 10.1063/1.4979514 28433024

[79] Tang Y-H, et al 2017. OpenRBC: a fast simulator of red blood cells at protein resolution. Biophys J. 112:2030–2037. 10.1016/j.bpj.2017.04.020 PMC5444005 28538143

[80] Zhang Y, Chai Z, Sun Y, Lykotrafitis G. 2020. A deep reinforcement learning model based on deterministic policy gradient for collective neural crest cell migration [preprint], arXiv, arXiv:2007.03190, https://arxiv.org/abs/2007.03190.

[81] Zhang Y, Chai Z, Lykotrafitis G. 2021. Deep reinforcement learning with a particle dynamics environment applied to emergency evacuation of a room with obstacles. Physica A. 571:125845.

[82] Toscano JD, Chai Z, Karniadakis GE. 2026. GRAFT-ATHENA: self-improving agentic teams for autonomous discovery and evolutionary numerical algorithms [preprint], arXiv, arXiv:2605.11117, https://arxiv.org/abs/2605.11117.

[83] Veillette A, Chen J. 2018. SIRPα-CD47 immune checkpoint blockade in anticancer therapy. Trends Immunol. 39:173–184. 10.1016/j.it.2017.12.005 29336991

[84] Wang S, Teng Y, Perdikaris P. 2021. Understanding and mitigating gradient flow pathologies in physics-informed neural networks. SIAM J Sci Comput. 43:A3055–A3081.

[85] Xiang Z, Peng W, Liu X, Yao W. 2022. Self-adaptive loss balanced physics-informed neural networks. Neurocomputing. 496:11–34.

[86] Wu C, Zhu M, Tan Q, Kartha Y, Lu L. 2023. A comprehensive study of non-adaptive and residual-based adaptive sampling for physics-informed neural networks. Comput Methods Appl Mech Eng. 403:115671.

[87] Monaco S, Apiletti D. 2023. Training physics-informed neural networks: one learning to rule them all? Results Eng. 18:101023.

[88] Yazdani A, Lu L, Raissi M, Karniadakis GE. 2020. Systems biology informed deep learning for inferring parameters and hidden dynamics. PLoS Comput Biol. 16:e1007575. 10.1371/journal.pcbi.1007575 PMC7710119 33206658

[89] Berendsen HJC, Postma JPM, Van Gunsteren WF, Dinola A, Haak JR. 1984. Molecular dynamics with coupling to an external bath. J Chem Phys. 81:3684–3690.

[90] Nosé S . 1984. A unified formulation of the constant temperature molecular dynamics methods. J Chem Phys. 81:511–519.

[91] Liu Z, et al 2024. KAN: Kolmogorov-Arnold networks [preprint], arXiv, arXiv:2404.19756, https://arxiv.org/abs/2404.19756.

[92] Sidharth SS, Keerthana AR, Gokul R, Anas KP. 2024. Chebyshev polynomial-based Kolmogorov-Arnold networks: an efficient architecture for nonlinear function approximation [preprint], arXiv, arXiv:2405.07200, https://arxiv.org/abs/2405.07200.

