# Supplementary material for: A multiscale signaling–biophysical framework reveals mechanisms of macrophage-mediated RBC clearance in sickle cell and Gaucher disease
Source: PNAS Nexus. 2026 Oct 6;5(10):pgag348. doi: 10.1093/pnasnexus/pgag348 (PMC13696960; doi:10.1093/pnasnexus/pgag348)
Supplement: Supplementary file 1 [file pgag348_supplementary_data.pdf]

Supplementary Information for

A Multiscale Signaling–Biophysical Framework Reveals Mechanisms of Macrophage-Mediated RBC Clearance in Sickle Cell and Gaucher Disease

Zhaojie Chai[1,*], Nazanin Ahmadi Daryakenari[2,*], and George E. Karniadakis[1,†]

[1]Division of Applied Mathematics, Brown University, Providence, Rhode Island, United States
[2]Center for Biomedical Engineering, School of Engineering, Brown University, Providence, Rhode Island, United States

[†]To whom correspondence should be addressed: george_karniadakis@brown.edu
[*]Zhaojie Chai and Nazanin Ahmadi Daryakenari contributed equally to this work.

This PDF file includes:

- Supplementary Text
- Figures S1 to S4
- Tables S1 to S11
- SI References

Table S1. Ordinary differential equations describing the intracellular signaling network governing RBC phagocytosis.

State	Governing equation
X_1	$\frac{dX_1}{dt} = -(K_7X_1X_{15} - K_{21}X_2) - (K_3K_6X_1X_{14} - K_3K_{20}X_{12})$
X_2	$\frac{dX_2}{dt} = (K_7X_1X_{15} - K_{21}X_2) - K_8X_2\left(1 - \frac{X_5}{X_5 + K_{23}}\right)^{K_{22}} + K_9X_3$
X_3	$\frac{dX_3}{dt} = K_8X_2\left(1 - \frac{X_5}{X_5 + K_{23}}\right)^{K_{22}} - K_9X_3$
X_4	$\frac{dX_4}{dt} = -K_{16}X_4X_3 + K_{17}X_5$
X_5	$\frac{dX_5}{dt} = K_{16}X_4X_3 - K_{17}X_5$
X_6	$\frac{dX_6}{dt} = K_{13}X_5X_7 - K_{12}X_6(X_{13} + X_{11} + X_{10})$
X_7	$\frac{dX_7}{dt} = -K_{13}X_5X_7 + K_{12}X_6(X_{13} + X_{11} + X_{10})$
X_8	$\frac{dX_8}{dt} = -K_1K_2K_{10}X_8 + K_{11}X_{10}$
X_9	$\frac{dX_9}{dt} = K_{15}X_{11} - K_4K_5K_{14}X_9$
X_{10}	$\frac{dX_{10}}{dt} = K_1K_2K_{10}X_8 - K_{11}X_{10}$
X_{11}	$\frac{dX_{11}}{dt} = -K_{15}X_{11} + K_4K_5K_{14}X_9$
X_{12}	$\frac{dX_{12}}{dt} = K_3K_6X_1X_{14} - K_3K_{20}X_{12} - K_{18}X_{12} + K_{19}X_{13}$
X_{13}	$\frac{dX_{13}}{dt} = K_{18}X_{12} - K_{19}X_{13}$
X_{14}	$\frac{dX_{14}}{dt} = -(K_3K_6X_1X_{14} - K_3K_{20}X_{12})$
X_{15}	$\frac{dX_{15}}{dt} = -(K_7X_1X_{15} - K_{21}X_2)$

Model parameters and initial conditions

The parameter set is summarized in Table S2, which specifies the nominal values used for forward simulation, identifies the parameters selected for inverse estimation, and provides their corresponding search ranges. The initial conditions employed in the forward problem are listed in Table S3. During the inverse analysis, only the designated unknown parameters were estimated. For parameters with available prior information, predefined search intervals were imposed as indicated in Table S2. The remaining estimated parameters were allowed to vary freely during optimization, while all other parameters were fixed at their nominal values.

Details of the identifiability analysis

Structural Identifiability Analysis

We performed a *structural identifiability analysis* to determine which parameters in the signaling ODE model can be uniquely inferred from the available observables. Following the rational-form strategy of (4), the governing equations were reformulated to ensure rational dependence on the

Table S2. Parameters of the signaling model, their nominal values, search ranges (when applicable), and biological descriptions.

**Parameter**	**Value**	**Unit**	**Search range**	**Description**
K_1	1.0×10^6	#/cell	Known	Band3 expression
K_2	1	#/cell	Known	Antibody
K_3	1	#/cell	Known	Matricellular protein TSP1
K_4	1	#/cell	Known	Phosphatidyl serine (PS)
K_5	1	unitless	Known	Erythrocyte rigidity
K_6	2.3×10^{-4}	$\#^{-1}$ s^{-1}	Known	Binding rate of CD47_alt to SIRPα
K_7	2.3×10^{-4}	$\#^{-1}$ s^{-1}	Known	Binding rate of CD47 to SIRPα
K_8	0.4	s^{-1}	0.3-0.5	Activation rate of CD47-bound SIRPα
K_9	0.02	s^{-1}	—	Deactivation rate of CD47-bound SIRPα
K_{10}	2.0×10^{-6}	s^{-1}	Known	Activation rate of Fc receptor
K_{11}	5.0×10^{-4}	s^{-1}	4.0×10^{-4}–6.0×10^{-4}	Deactivation rate of Fc receptor
K_{12}	5.2×10^{-7}	s^{-1}	Known	Activation rate of Myosin IIa
K_{13}	3.8×10^{-8}	s^{-1}	Known	Deactivation rate of Myosin IIa
K_{14}	0.51	s^{-1}	—	Activation rate of PS receptor
K_{15}	1.0×10^{-3}	s^{-1}	—	Deactivation rate of PS receptor
K_{16}	0.007	s^{-1}	—	Activation rate of SHP1
K_{17}	0.002	s^{-1}	—	Deactivation rate of SHP1
K_{18}	0.001	s^{-1}	Known	Activation rate of TSP1-bound SIRPα
K_{19}	0.03	s^{-1}	Known	Deactivation rate of TSP1-bound SIRPα
K_{20}	0.4	s^{-1}	Known	Dissociation rate of CD47_alt from SIRPα
K_{21}	0.4	s^{-1}	Known	Dissociation rate of CD47 from SIRPα
K_{22}	3	unitless	Known	Hill coefficient of SHP–SIRPα activation
K_{23}	6.0×10^4	#	5.8×10^4–6.2×10^4	K_d coefficient of SHP–SIRPα activation

Table S3. Initial conditions of the signaling model. * Parameter obtained from fitting; ** Phenomenological parameters; *** Macrophage receptor levels.

**Species**	**Variable**	**Initial value**	**Unit**	**Description**
SIRPα	X_1	3.0×10^3 ***	#/cell	SIRPα receptor
CD47-SIRPα	X_2	0	#/cell	CD47-bound SIRPα receptor
CD47-SIRPα_act	X_3	0	#/cell	Activated CD47-bound SIRPα receptor
SHP1_inact	X_4	2.8×10^6	#/cell	Inactive SHP1
SHP1_act	X_5	0	#/cell	Activated SHP1
Myo2a_inact	X_6	2200	#/cell	Inactive myosin IIa
Myo2a_act	X_7	0	#/cell	Activated myosin IIa
FCR	X_8	5.0×10^4 ***	#/cell	Fc receptor expression
PSR	X_9	1.5×10^4 ***	#/cell	Phosphatidylserine receptor
FCR_act	X_{10}	0	#/cell	Activated Fc receptor
PSR_act	X_{11}	0	#/cell	Activated PS receptor
TSP1.CD47-SIRPα	X_{12}	1	#/cell	TSP1-bound complex
TSP1.CD47-SIRPα_act	X_{13}	1	#/cell	Activated TSP1-bound complex
CD47_alt	X_{14}	1	#/cell	Conformationally altered CD47
CD47	X_{15}	2.0×10^4	#/cell	CD47 expression

Table S4. Comparison of simulated and experimental diffusion coefficients. Physical values are obtained using the DPD unit mapping from Table S9.

Sample	D_{DPD}	D_{phys} (cm^2/s)
Green Line	1.46×10^{-3}	1.05×10^{-7}
Blue Line	5.07×10^{-4}	3.66×10^{-8}
Black Line	1.83×10^{-4}	1.32×10^{-8}
IgG (experimental) (8, 21, 33)	—	10^{-8}–10^{-6}

unknown parameters. In particular, the second and third equations contain the term

$$g(t) = \left(\frac{K_{23}}{X_5 + K_{23}}\right)^{K_{22}},$$

where K_{22} appears as an exponent. Because the algorithms implemented in `StructuralIdentifiability.jl` require rational parameter dependence, this transcendental form cannot be analyzed directly. Therefore, K_{22} was fixed to its experimentally determined value ($K_{22} = 3$) and treated as known during the identifiability analysis. Two analysis scenarios were considered: (i) all kinetic and affinity parameters treated as unknown except K_{22}, and (ii) a reduced set in which experimentally measurable parameters were fixed. Parameters were classified as *globally identifiable*, *locally identifiable*, *nonidentifiable*, or *fixed* based on the symbolic analysis results.

Practical Identifiability Analysis

Practical identifiability was evaluated to determine the recoverability of model parameters under finite and noisy measurements (24, 32). To mimic experimental variability, multiplicative noise was introduced into observable states at three levels (1%, 5%, and 10%) using independent uniform perturbations. No artificial noise was added to the experimentally measured CD47–SIRPα activation state (X_3). Based on the results of the structural identifiability analysis, a subset of identifiable kinetic parameters was estimated using nonlinear least-squares optimization in log-parameter

Table S5. Summary of structural identifiability results for the signaling model parameters under three scenarios: (i) all parameters treated as unknown, (ii) selected parameters fixed based on available experimental data, and (iii) additional nonidentifiable parameters fixed according to prior biological knowledge. "**Known***" indicates parameter K_{22}, which was fixed to its experimentally determined value (see Structural Identifiability Analysis for details).

Parameter	Description	All unknown	Known exp. params	Fixed NI params
K1	Band3	Nonidentifiable	Nonidentifiable	Known
K2	Antibody	Nonidentifiable	Known	Known
K3	TSP1	Nonidentifiable	Known	Known
K4	PS	Nonidentifiable	Known	Known
K5	Rigidity	Nonidentifiable	Known	Known
K6	$K_f^{\text{CD47altSIRPa}}$	Nonidentifiable	Known	Known
K7	$K_f^{\text{CD47SIRPa}}$	Globally identifiable	Known	Known
K8	$K_f^{\text{CD47SIRPaact}}$	Globally identifiable	Globally identifiable	Globally identifiable
K9	$K_f^{\text{CD47SIRPainact}}$	Globally identifiable	Globally identifiable	Globally identifiable
K10	K_f^{FCRact}	Nonidentifiable	Nonidentifiable	Known
K11	K_f^{FCRinact}	Globally identifiable	Globally identifiable	Globally identifiable
K12	K_f^{myo2aact}	Nonidentifiable	Nonidentifiable	Known
K13	$K_f^{\text{myo2ainact}}$	Nonidentifiable	Nonidentifiable	Known
K14	K_f^{PSRact}	Nonidentifiable	Globally identifiable	Globally identifiable
K15	K_f^{PSRinact}	Globally identifiable	Globally identifiable	Globally identifiable
K16	K_f^{SHP1act}	Globally identifiable	Globally identifiable	Globally identifiable
K17	$K_f^{\text{SHP1inact}}$	Globally identifiable	Globally identifiable	Globally identifiable
K18	$K_f^{\text{TSP1CD47/SIRPaact}}$	Globally identifiable	Known	Known
K19	$K_f^{\text{TSP1CD47/SIRPainact}}$	Globally identifiable	Known	Known
K20	$K_r^{\text{CD47altSIRPa}}$	Nonidentifiable	Known	Known
K21	$K_r^{\text{CD47SIRPa}}$	Globally identifiable	Known	Known
K22	SHP_hillcoeff	**Known***	**Known***	**Known***
K23	SHP_kd	Globally identifiable	Globally identifiable	Globally identifiable

space, which ensures parameter positivity and improves numerical stability. The objective function was defined as a weighted sum of squared residuals between model predictions and observations, normalized by the magnitude of each state variable.

Local practical identifiability was first assessed using the Fisher Information Matrix (FIM), which was computed from the sensitivity matrix of model outputs with respect to the unknown parameters (24, 32). The resulting matrix was used to evaluate parameter variances, parameter correlations, and the conditioning of the estimation problem.

To complement the local FIM-based analysis, profile likelihood analysis was performed to examine parameter identifiability more globally. In this procedure, each parameter was fixed sequentially across a range of values in log-space while the remaining parameters were re-optimized (24). The increase in the objective function was then compared with the χ^2 threshold corresponding to a 95% confidence level. Parameters exhibiting finite profile widths were considered practically identifiable, whereas flat or unbounded profiles indicated non-identifiability.

Dissipative particle dynamics (DPD) method and RBC model

Dissipative particle dynamics (DPD) method

In this work, we build our coarse-grained macrophage model and investigate the active phagocytosis process with the dissipative particle dynamics (DPD) method, a mesoscopic particle-based method that has been widely used for problems in complex fluids and soft matter (5, 9, 10, 14). In a

Table S6. Summary of practical identifiability results across observational noise levels. FIM σ denotes the relative standard deviation estimated from the inverse Fisher Information Matrix. PL indicates whether the profile likelihood exceeded the 95% confidence threshold. Agreement reflects consistency between FIM and profile likelihood conclusions.

Noise	Parameter	Fitted Value	FIM σ	PL Identifiable	Agreement
1%	K_8	3.55e-01	14.77	No	Yes
	K_9	2.92e-02	0.43	Yes	Yes
	K_{11}	4.85e-04	124.69	No	Yes
	K_{14}	5.17e-01	0.60	Yes	Partial
	K_{15}	9.76e-04	22.55	No	Yes
	K_{16}	1.17e-02	0.48	Yes	Yes
	K_{17}	1.99e-03	1.55	No	Yes
	K_{23}	4.58e+04	9.34	Yes	Partial
5%	K_8	3.08e-01	13.24	Yes	Partial
	K_9	2.46e-02	0.37	Yes	Yes
	K_{11}	5.00e-04	107.62	No	Yes
	K_{14}	5.40e-01	0.62	Yes	Partial
	K_{15}	1.00e-03	23.19	No	Yes
	K_{16}	9.48e-03	0.44	Yes	Yes
	K_{17}	2.01e-03	1.64	Yes	Partial
	K_{23}	4.36e+04	8.48	Yes	Partial
10%	K_8	2.06e-01	7.73	Yes	Partial
	K_9	3.16e-02	0.51	Yes	Partial
	K_{11}	3.58e-04	62.26	No	Yes
	K_{14}	8.82e-01	0.80	Yes	Partial
	K_{15}	3.53e-04	68.68	No	Yes
	K_{16}	1.26e-02	0.49	Yes	Yes
	K_{17}	3.89e-03	0.99	Yes	Partial
	K_{23}	7.74e+04	5.20	Yes	Partial

Table S7. Comparison of PINNs parameter estimation with and without search space constraints. Search limits were applied to parameters identified as weakly constrained in the practical identifiability analysis (red). Constraining the search space reduces variance and improves stability without affecting well-identified parameters.

Parameter	True Value	Without Limit			With Limit		
		Mean	Std	Median	Mean	Std	Median
K8	4.00e-1	7.16e-1	1.42e-1	7.74e-1	3.83e-1	3.37e-2	3.7e-1
K9	2.00e-2	1.74e-2	4.39e-4	1.75e-2	1.98e-2	3.49e-4	1.97e-2
K11	5.00e-4	1.24e-3	7.02e-4	8.90e-4	5.15e-4	9.00e-6	5.10e-4
K14	5.10e-1	5.09e-1	3.71e-3	5.10e-1	5.21e-1	2.71e-4	5.21e-1
K15	1.00e-3	9.12e-4	2.43e-4	1.00e-3	1.11e-3	3.3e-5	1.10e-3
K16	7.00e-3	6.92e-3	1.42e-4	6.90e-3	6.88e-3	3.60e-5	6.87e-3
K17	2.00e-3	1.95e-3	1.08e-4	1.97e-3	1.94e-3	2.20e-5	1.93e-3
K23	6.00e+4	4.10e+4	5.82e+3	4.23e+4	5.92e+4	1.41e+3	5.85e+4

DPD simulation, a group of atoms is represented by a bead at its center, which greatly reduces the computational cost. The interactions between two coarse-grained beads i and j are integrated into three types of forces:

$$\mathbf{F}_i = \sum_{j \neq i} (\mathbf{F}_{ij}^{\mathrm{C}} + \mathbf{F}_{ij}^{\mathrm{D}} + \mathbf{F}_{ij}^{\mathrm{R}}), \tag{S1}$$

Table S8. Relative error (%) of inferred parameters under different noise levels for PINNs and tanh-cPIKANs.

	PINNs								Tanh-cPIKANs							
**Noise**	**K8**	**K9**	**K11**	**K14**	**K15**	**K16**	**K17**	**K23**	**K8**	**K9**	**K11**	**K14**	**K15**	**K16**	**K17**	**K23**
0%	7.82	1.86	0.76	0.05	0.18	1.99	2.65	2.43	7.16	2.42	12.50	0.63	5.17	1.44	0.73	1.92
5%	7.96	2.06	2.43	2.22	1.01	1.78	2.98	2.27	8.80	2.61	13.11	1.25	5.78	1.73	1.10	2.30
10%	9.90	7.00	0.89	1.95	0.54	2.71	5.64	1.15	9.50	3.09	12.82	1.50	5.94	2.19	1.47	2.73

where $\mathbf{F}_{ij}^{\mathrm{C}}$, $\mathbf{F}_{ij}^{\mathrm{D}}$ and $\mathbf{F}_{ij}^{\mathrm{R}}$ are conservative, dissipative and random forces, respectively. The typical forms of these three pairwise forces are described as follows:

$$\mathbf{F}_{ij}^{\mathrm{C}} = a_{ij}\omega_{\mathrm{C}}(r_{ij})\mathbf{e}_{ij} \ , \tag{S2}$$

$$\mathbf{F}_{ij}^{\mathrm{D}} = -\gamma_{ij}\omega_{\mathrm{D}}(r_{ij})(\mathbf{e}_{ij}\cdot\mathbf{v}_{ij})\mathbf{e}_{ij} \ , \tag{S3}$$

$$\mathbf{F}_{ij}^{\mathrm{R}} = \sigma_{ij}\omega_{\mathrm{R}}(r_{ij})\xi_{ij}\Delta t^{-1/2}\mathbf{e}_{ij} \ , \tag{S4}$$

where r_{ij} is the distance between beads i and j, $\mathbf{e}_{ij}$ is the corresponding unit vector, and ξ_{ij} is Gaussian white noise with a mean of zero and unit variance. a_{ij} determines the strength of the conservative force, which we set to 6.0 in our simulations. γ_{ij} and σ_{ij} reflect the strength of the dissipative and random forces, respectively, and $\omega_{\mathrm{C}}(r_{ij})$, $\omega_{\mathrm{D}}(r_{ij})$, and $\omega_{\mathrm{R}}(r_{ij})$ are the corresponding weighting functions. To maintain the energy conservation of the simulation system and satisfy the fluctuation-dissipation theorem, two further conditions must be enforced:

$$\sigma_{ij}^2 = 2\gamma_{ij}k_{\mathrm{B}}T, \tag{S5}$$

$$\omega_{\mathrm{D}}(r_{ij}) = [\omega_{\mathrm{R}}(r_{ij})]^2 = (1 - r_{ij}/r_c)^{2k}, \tag{S6}$$

where k_{B} is the Boltzmann constant and T is the temperature. Additionally, r_c is the cutoff radius and k is the exponent for the DPD envelope.

RBC model

To investigate the role of each biophysical factor in the blood viscosity and, ultimately, Gaucher Disease RBCs computationally, we need to model the individual RBCs accurately, without compromising the essential hydrodynamics of the system and the convective transport processes that govern the blood flow. An RBC has a highly deformable biconcave nucleus-free membrane with a diameter of $\sim$8 μm. Known as a viscoelastic object, the RBC shows both liquid-like (viscous) and solid-like (elastic) responses to an applied deformation at the same time. This allows RBCs to deform sufficiently to squeeze through capillaries as small as 3 μm, preserve their shapes at small deformation rates, and orient in the flow direction at larger deformation rates in wider arteries. The RBC membrane is modeled as a set of N_ν DPD particles with the three-dimensional coordinates X_i ($i \in 1, \ldots, N_\nu$) in a triangulated network of springs with a dashpot on the surface. To model the incompressibility of the RBC membrane, area and volume constraints are applied. By incorporating bending resistance between neighboring triangles, the bending rigidity of the membrane is captured. In this work, the DPD and fluid membrane parameters used in the phagocytosis simulations are summarized in Table S9. The shear modulus and bending modulus of normal RBCs in our work are selected to be $E_{s_0} = 4.792\,\mu$N/m, $E_{b_0} = 2.9 \times 10^{-19}$ J (7).

DPD Model Framework

In our simulations, the macrophage membrane is represented as a deformable layer composed of DPD beads, with embedded receptor slots corresponding to SIRPα molecules. IgG antibodies are modeled as flexible, coarse-grained beads that can bind membrane receptors. Antibody–receptor interactions are implemented through attractive interparticle potentials or reactive binding rules between IgG beads and SIRPα receptors. Antibody diffusion is explicitly simulated both in the 3D extracellular solution and laterally along the membrane surface after binding. These simulation outputs are used to calculate the effective SIRPα activation state, X_3, because CD47–SIRPα binding is the upstream trigger of the inhibitory signaling cascade, the DPD-derived receptor occupancy provides a mechanistic proxy for the activation level of the signaling complex represented by X3. In the signaling model, X_3 represents the activated CD47-SIRPα receptor complex. In the presence of anti-SIRPα antibodies, receptor occupancy reduces the availability of SIRPα for CD47 binding, thereby decreasing the effective activation level of X_3. For anti-CD47 antibodies, X_3 is inferred based on the remaining CD47 availability for SIRPα binding—consistent with observed increases in phagocytic activity when the SIRPα–CD47 interaction is blocked (35).

Signaling Interaction Model

To characterize antibody-mediated modulation of macrophage signaling, we modeled the binding interactions between antibodies and membrane receptors such as SIRPα using DPD (6). In this framework, antibodies were represented as diffusing particles in the extracellular domain, capable of binding to discrete receptor sites distributed on the macrophage membrane. Binding was governed by a short-range attractive Morse potential between antibodies and receptor vertices:

$$V(r) = D_e\big(e^{-2\beta(r-r_0)} - 2e^{-\beta(r-r_0)}\big) \tag{S7}$$

where r is the distance between an antibody and a receptor particle, D_e is the well depth (binding strength), β controls the interaction range, and r_0 is the equilibrium binding distance. Once bound, the lateral mobility of antibodies was restricted by an additional harmonic potential to mimic membrane anchoring, allowing us to capture post-binding diffusion and clustering.

The spatial layout of receptor sites was initialized with non-overlapping positions across the membrane surface. Receptor occupancy over time was recorded as the fraction of bound receptors, denoted $X_3(t)$, and was extracted directly from the DPD simulations under different molecular crowding and antibody concentration conditions. The resulting $X_3(t)$ profiles were interpolated and used as input or constraints in the PINNs-based ODE model of CD47-SIRPα signaling. This hybrid DPD-ODE approach links antibody binding dynamics to macrophage activation states and enables evaluation of immunomodulatory strategies targeting the CD47-SIRPα axis (22).

DPD Simulation Parameters for Antibody–Receptor Binding

Particle Types and Initial Configuration

Due to the limitations of experimental *in vitro* measurements mentioned earlier, we employed computational approaches. Advances in computational power over the past two decades have stimulated the development and application of multiscale biophysical models for studying complex cellular systems (2, 3, 23, 27). In parallel, machine learning approaches have increasingly been applied to collective cell dynamics and particle-based simulations (28, 38, 39). A coarse-grained Dissipative

Particle Dynamics (DPD) model was employed to simulate antibody–receptor interactions occurring on the macrophage membrane. The simulated system consisted of three main particle types representing the extracellular environment, antibodies, and membrane structures. Solvent particles were used to represent the surrounding extracellular fluid and served as a thermalized background that mediates hydrodynamic interactions between all components of the system. Antibodies, such as anti-SIRPα IgG molecules, were modeled as spherical DPD beads with an effective diameter of $\sigma = 1.0\ \mu m$. These particles were initially distributed randomly throughout the solvent volume and allowed to diffuse freely until encountering receptor sites on the macrophage surface.

The macrophage membrane was represented as a triangulated network of DPD particles forming a deformable surface. Receptor sites corresponding to SIRPα molecules were implemented as a subset of these membrane particles. These receptors were arranged in predefined spatial clusters to mimic the presence of lipid raft–like membrane domains that locally enrich receptor density. In the initial configuration, 300 antibody particles were uniformly distributed within the bulk solvent domain. The macrophage membrane consisted of approximately 1000 DPD beads forming a spherical cap geometry, among which 200 particles were designated as receptor sites. Receptor clusters occupied roughly 30% of the membrane area in order to reproduce experimentally observed membrane microdomain organization.

Interaction Potentials

Binding interactions between antibody particles and SIRPα receptors were modeled using a Morse potential. To prevent unphysical particle overlap and to maintain the hydrodynamic consistency of the system, standard DPD conservative, dissipative, and random forces were applied between all particle pairs. The conservative force followed the standard soft repulsive form

$$F_{ij}^{C} = a_{ij} \left(1 - \frac{r_{ij}}{r_c}\right) \hat{r}_{ij}, \qquad r_{ij} < r_c, \tag{S8}$$

with a maximum repulsion parameter $a_{ij} = 25$ and a cutoff radius $r_c = 1.0\,\sigma$. After binding occurs, antibody particles are additionally connected to receptor sites through a harmonic spring potential to represent the reduced lateral mobility of membrane-bound complexes.

Simulation Domain and Conditions

The simulation domain had dimensions of $60 \times 60 \times 50\ \mu m^3$. Periodic boundary conditions were imposed along the x and z directions, while reflective boundaries were applied along the y direction to mimic confinement near the membrane interface. The simulations were typically run for 4×10^6 to 8×10^6 time steps using a modified velocity–Verlet integration scheme combined with a DPD thermostat to maintain the system temperature.

Computational cost

All simulations were performed using an extended version of the code developed in LAMMPS. Each simulation takes about 4,000,000 time steps to 8,000,000 time steps. A typical simulation requires 1200 CPU core hours to 2400 CPU core hours by using the computational resources (Intel Xeon E5-2670 2.6 GHz 24-core processors) at the Center for Computation and Visualization at Brown University.

Estimation and Validation of Diffusion Coefficients

In the diffusive regime, the MSD grows linearly with time according to the Einstein relation for three-dimensional Brownian motion,

$$\mathrm{MSD}(t) = 6D_{\mathrm{eff}}t, \tag{S9}$$

where D_{eff} denotes the effective diffusion coefficient. Linear regression was therefore applied to the long-time portion of the MSD curves, and the slope divided by six to obtain the diffusion coefficient in DPD units, D_{DPD}. In addition, Figure S1D shows the statistical distribution of diffusion coefficients estimated from individual particle trajectories. To convert the simulated diffusion coefficients to physical units, we employed the unit mapping of the DPD model (Table S9). The characteristic length and time scales are $L^* = 1.0 \times 10^{-6}\,\mathrm{m}$ and $t^* = 1.386 \times 10^{-4}\,\mathrm{s}$, which yield the conversion factor

$$\frac{(L^*)^2}{t^*} = 7.22 \times 10^{-9}\,\mathrm{m}^2/\mathrm{s} = 7.22 \times 10^{-5}\,\mathrm{cm}^2/\mathrm{s}.$$

Accordingly, the physical diffusion coefficient is obtained as

$$D_{\mathrm{phys}} = D_{\mathrm{DPD}}\frac{(L^*)^2}{t^*}. \tag{S10}$$

Using this mapping, the diffusion coefficients extracted from the MSD curves were converted to physical units. For example, the green trajectory in Figure S1C yields $D_{\mathrm{DPD}} = 1.46 \times 10^{-3}$, corresponding to $D_{\mathrm{phys}} = 1.05 \times 10^{-7}\,\mathrm{cm}^2/\mathrm{s}$. The full set of estimated diffusion coefficients is summarized in Table S4.

The simulated values fall within the experimentally reported diffusion range for IgG antibodies (10^{-8}–$10^{-6}\,\mathrm{cm}^2/\mathrm{s}$) (8, 21, 33). This validation is particularly relevant for modeling antibody-mediated processes in diseases such as GD, which is characterized by elevated levels of circulating IgG autoantibodies (including IgG1, IgG2, and IgG3), although these are directed against the accumulated substrate rather than against the RBC surface (20). In particular, the higher diffusivity observed for the green and blue trajectories reflects the smaller effective particle sizes represented in the coarse-grained DPD model, whereas the black trajectory provides a lower bound consistent with restricted transport in crowded environments. The overall agreement with experimental measurements supports the validity of the DPD framework for capturing mesoscale antibody diffusion.

To interpret the coarse-grained representation in molecular terms, we estimated the number of physical molecules represented by each DPD particle using the concentration mapping defined in Table S9. The concentration unit was chosen as $C^* = 1.0\,\mathrm{nM}$, corresponding to a number density $n^* = C^*V^*/\rho$, where $V^* = (L^*)^3$ is the volume unit and ρ is the particle number density. For an antibody-like species initialized at $C_0 = 1\,\mathrm{nM}$ ($10^{-3}\,\mathrm{mol/m}^3$), the number of moles represented by each DPD particle is approximately

$$n = \frac{C_0V^*}{\rho} \approx 5.0 \times 10^{-23}\,\mathrm{mol}.$$

Multiplying by Avogadro's number yields approximately 30 molecules per DPD particle. Thus, each coarse-grained particle in the simulation effectively represents $\mathcal{O}(10^1)$ antibody molecules, providing a physically interpretable mapping between the DPD representation and the underlying molecular population.

Validation of Antibody Dynamics

As shown in Figure S2A, our simulation predicts a time-resolved receptor occupancy curve where the activation of the CD47–SIRPα complex (X_3) reaches a plateau of approximately 70% of the healthy baseline (41). This simulated 30% reduction is consistent with experimental observations indicating a diminished "don't eat me" signal in RBCs, providing a quantitative basis to evaluate how CD47 depletion or receptor blockade impacts inhibitory signaling activity (12).

The binding kinetics extracted from the early-time regime correspond to an effective on-rate of 1.2×10^5 $M^{-1}s^{-1}$, comparable to surface plasmon resonance measurements of CD47–SIRPα binding, which fall in the range of 10^5–10^6 $M^{-1}s^{-1}$ (30). Figure S2B depicts the spatial distribution of antibody–receptor complexes across the macrophage membrane. The simulations establish a quantitative framework for analyzing diffusion and localized binding dynamics. The results are consistent with fluorescence microscopy observations, where receptor–ligand complexes organize into microdomains with elevated local densities (29). While these results support the biological relevance of the model, it is important to note that the current framework does not include an explicit cytoskeletal scaffold. Such structures are known to restrict receptor diffusion and organize membrane domains in RBCs and neurons (2, 3, 15, 16, 40), and their absence may contribute to quantitative differences between simulation and experiment.

Structural and Practical Identifiability Analysis

When all parameters were treated as unknown, several kinetic rates were found to be globally identifiable, including K_7, K_8, K_9, K_{11}, K_{15}, K_{16}, K_{17}, K_{18}, K_{19}, K_{21}, and K_{23}. These parameters are structurally recoverable from perfect measurements and therefore form the core set of candidates for subsequent inverse analysis. In contrast, parameters associated with membrane composition, rigidity, alternative receptor binding pathways, and certain cytoskeletal activation steps, such as K_1–K_6, K_{10}, K_{12}, K_{13}, and K_{20}, were determined to be structurally nonidentifiable. Their nonidentifiability arises from model symmetries and compensatory transformations that leave the observable outputs invariant.

Incorporating experimentally known parameters into the analysis significantly improved identifiability of specific reaction rates. In particular, fixing biologically well-characterized quantities (e.g., ligand concentrations or measured kinetic constants) resolved several previously nonidentifiable directions in parameter space. Under this partially constrained setting, additional parameters, including K_{14}, became globally identifiable. This demonstrates that identifiability in nonlinear biochemical networks depends strongly on prior knowledge and observable selection.

In the final reduced scenario, parameters classified as structurally nonidentifiable and lacking sufficient experimental constraints were fixed to representative literature values. This identifiability-informed reduction eliminates symmetry-induced ambiguities and removes flat directions from the parameter space. The resulting reduced parameter set—comprising K_8, K_9, K_{11}, K_{14}, K_{15}, K_{16}, K_{17}, and K_{23}—forms the structurally identifiable subset used in subsequent inverse modeling and PIN-based parameter estimation.

Notably, the condition number decreased slightly at the highest noise level (10%), reflecting changes in local curvature near the perturbed optimum rather than improved identifiability. Strong positive coupling is consistently observed between K_8 and K_{23}, as well as between K_9 and K_{16}, while K_8 and K_{16} exhibit pronounced negative correlation. These dominant dependencies persist under increasing noise, indicating intrinsic parameter coupling in the model structure rather than noise-induced artifacts. In contrast, parameters such as K_{11} and K_{15} do not display strong pairwise correlations but instead exhibit weak curvature signatures in the local sensitivity landscape.

This weak curvature indicates that the objective function varies only marginally with respect to these parameters near the optimum, which results in broad, shallow minima and large uncertainty estimates. Such flat directions in the loss surface are consistent with their flat profile likelihoods and confirm their practical non-identifiability despite structural identifiability.

Profile likelihood analysis provides complementary global insight. At 1% noise, parameters K_9, K_{14}, K_{16}, and K_{23} exhibit finite confidence intervals exceeding the 95% χ^2 threshold, indicating practical identifiability. In contrast, K_{11}, K_{15}, and K_{17} fail to cross the threshold, demonstrating flat likelihood profiles and non-identifiability. At 5% noise, a similar pattern is observed, with K_8 becoming identifiable while K_{11} and K_{15} remain unidentifiable. At 10% noise, K_8, K_9, K_{14}, K_{16}, K_{17}, and K_{23} retain identifiable profiles, whereas K_{11} and K_{15} persistently exhibit flat profiles with effectively unbounded confidence intervals.

Parameters K_{16} and K_9 consistently demonstrate strong curvature and bounded likelihood profiles across noise levels that indicate robust recoverability. Conversely, K_{11} and K_{15} exhibit large FIM-derived variance estimates and flat profile likelihoods, confirming practical non-identifiability despite being structurally identifiable. For practical identifiability assessment, two complementary criteria were used. The FIM was computed in log-parameter space, and uncertainty was quantified as $\sigma_{\text{FIM}} = \sqrt{(FIM^{-1})_{ii}}$. Parameters with $\sigma_{\text{FIM}} < 0.5$ were classified as locally identifiable, while parameters with $\sigma_{\text{FIM}} \geq 0.5$ were considered weakly constrained. Because σ_{FIM} is defined in log space, this threshold corresponds approximately to less than 65% multiplicative uncertainty in parameter magnitude. Profile likelihood identifiability was evaluated using the 95% χ^2 threshold for one degree of freedom. Since the objective function was defined as $\frac{1}{2}\sum r_i^2$, the corresponding cutoff for practical identifiability was $\Delta\mathcal{L} > \chi^2_{0.95,1}/2 = 1.92$. Agreement between FIM and profile likelihood was defined as consistency between these two classifications. We demonstrate that parameter identifiability is constrained by strong correlations predicted by the FIM and confirmed via Monte Carlo sampling (Figure S4).

Comparison of q-Scheduling Methods

An additional component of the modeling framework involved a q-scheduling strategy designed to improve the convergence and stability of PINNs. In this formulation, the parameter q determines the relative weight between the physics-based loss term and the data-based loss term in the composite training objective. Gradually increasing the contribution of the physics loss during training allows the neural network to first approximate the available experimental data before enforcing the full system of governing equations. This approach is conceptually analogous to temperature scheduling methods used in thermostats such as Berendsen or Nosé–Hoover, which regulate the rate at which systems approach equilibrium (31). By preventing stiff ODE residuals from dominating the early stages of training, the scheduling strategy reduces the risk of convergence to poor local minima and improves the stability of parameter inference. Comparative tests across different scheduling approaches indicated that smooth and continuous q-adjustment generally yields better training stability and prediction accuracy than stepwise schemes (36).

In addition to loss weighting, we explored how the number and distribution of residual points affect the performance of PINNs in inverse parameter estimation. We compared both fixed and randomized residual sampling strategies across a range of residual point counts. Our results show that model accuracy is highly sensitive to this configuration: using a moderate number of residual points—neither too sparse nor overly dense—leads to significantly improved parameter recovery. Furthermore, randomized sampling consistently outperformed fixed sampling at comparable point counts, likely due to better generalization and reduced bias in residual enforcement. This behavior is consistent with recent work showing that residual point refinement and adaptive sampling act

Table S9. DPD and fluid membrane parameters in phagocytosis simulations.

Parameter	Symbol	Value used
strength of the conservative force	a_{ij}	6
strength of the dissipative force	γ_{ij}	15
strength of the random force	σ_{ij}	2.63
cutoff distance of DPD interaction	r_c	2.7
exponent of DPD interaction	k	0.5
temperature	$k_{\mathrm{B}}T$	0.23
energy unit of fluid membrane interaction	ε	1.0
length unit of fluid membrane interaction	σ	1.0
cutoff distance of fluid membrane interaction	r_{cut}	2.6
exponent of fluid membrane interaction	ζ	4
angular coefficient of fluid membrane interaction	μ	3
spontaneous angle of fluid membrane interaction	$\sin\theta_0$	0

as a form of curriculum learning, guiding the network toward physically consistent solutions while avoiding optimization instability (18, 34). These findings emphasize that residual point design is a key component in building robust PINNs architectures for biological inference problems.

We systematically evaluated different q-scheduling strategies and their impact on PINNs parameter inference accuracy. The parameter q controls the relative weight of the physics-based loss term during training, and scheduling its evolution over iterations has been proposed as a way to stabilize optimization and improve convergence (31, 37). Figure S3, panels A and B, show the evolution of q as a function of iteration for several scheduling strategies, targeting final values of $q = 2$ in panel A and $q = 10$ in panel B. Among the methods tested, the Berendsen-type schedules (1) and the Nose–Hoover-inspired schedule (19) achieve smooth and gradual increases in q, whereas the step method exhibits abrupt jumps. Smooth convergence of q is desirable because it helps avoid the premature dominance of the physics loss and allows the model to fit the sparse data during the early stages of training (11). The baseline case ($q = 1$, with no warm-up) exhibited relatively poor inference performance, and step scheduling likewise showed unfavorable behavior across the tested target q values. By contrast, Berendsen-based and Nose–Hoover schedules yielded qualitatively better results overall. In particular, the Berendsen-type methods displayed the most favorable performance and consistently outperformed both the linear and step strategies at $q = 2$ and $q = 10$. Collectively, these observations suggest that thermostat-inspired warm-up schedules can improve training stability and support better identifiability.

It is noteworthy that the step scheduling results are identical for $q = 2$ and $q = 10$. This is because, in both cases, the model selected the best checkpoint at approximately 18,000 iterations—before the scheduled step increase in q took effect. Consequently, the later jump in q inherent to the step method had no impact on the final reported results. This observation highlights a potential drawback of step scheduling: its delayed activation of the physics loss can lead the model to overfit the early data-fitting phase and ignore the later dynamics. These results highlight the importance of warm-up scheduling when employing PINNs for inverse problems with stiff physics constraints. In particular, Berendsen-type q-scheduling provides a practical and effective approach to mitigate training pathologies and enhance inference accuracy (11, 37).

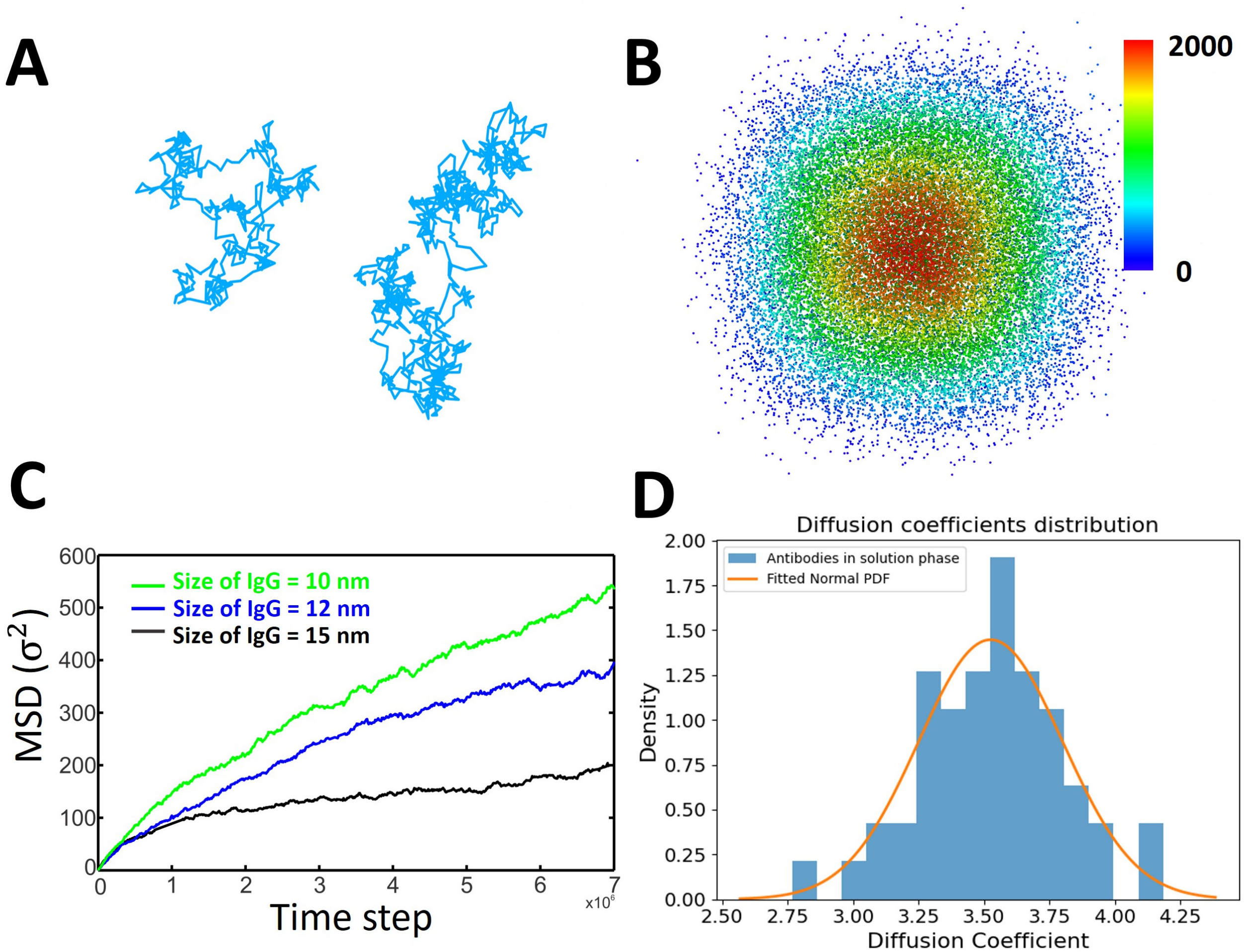

Figure S1. Validation of diffusive behavior in DPD simulations. (A) Sample trajectories of selected particles undergoing diffusion in 3D space, demonstrating characteristic random-walk behavior. (B) Radial particle density distribution centered at the origin, with color mapping from blue (low) to red (high) density, showing isotropic and spherically symmetric spreading consistent with Brownian diffusion. (C) Mean squared displacement (MSD) of IgG of different sizes (10, 12, and 15 nm), where smaller IgG exhibit faster diffusion as indicated by steeper MSD curves. (D) Distribution of diffusion coefficients for IgG in the solution phase (blue histogram), fitted with a normal probability density function (orange line), illustrating variability in antibody mobility across the ensemble.

Physics-Informed Neural Networks Architecture

In the baseline PINNs model, the network is a fully connected feed-forward neural network with tanh activation functions. For an input t, the forward propagation through an L-layer network is given by

$$\mathbf{z}^{(0)} = t, \tag{S11}$$

$$\mathbf{z}^{(\ell)} = \tanh\Big(W^{(\ell)}\mathbf{z}^{(\ell-1)} + \mathbf{b}^{(\ell)}\Big), \quad \ell = 1, \ldots, L, \tag{S12}$$

$$\hat{\mathbf{X}}(t) = W^{(L+1)}\mathbf{z}^{(L)} + \mathbf{b}^{(L+1)}, \tag{S13}$$

where $W^{(\ell)}$ and $\mathbf{b}^{(\ell)}$ denote the weight matrices and bias vectors of layer ℓ. The final layer is linear to allow unrestricted output scaling.

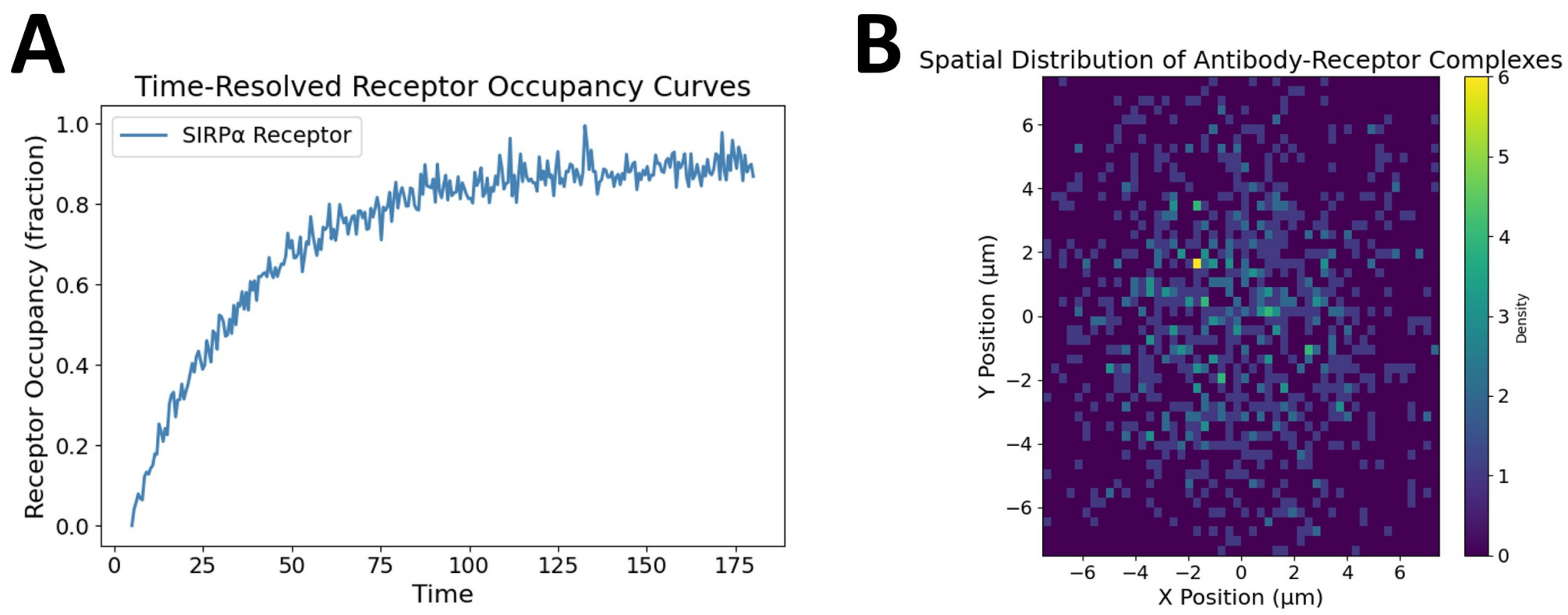

Figure S2. Simulation-derived observables characterizing IgG antibody dynamics and membrane engagement. (A) Time-resolved receptor occupancy curve for anti-SIRPα IgG, showing progressive binding to SIRPα receptors on the macrophage membrane. (B) Spatial distribution heatmap of antibody–receptor complexes on the membrane, illustrating clustering due to lateral diffusion and binding kinetics.

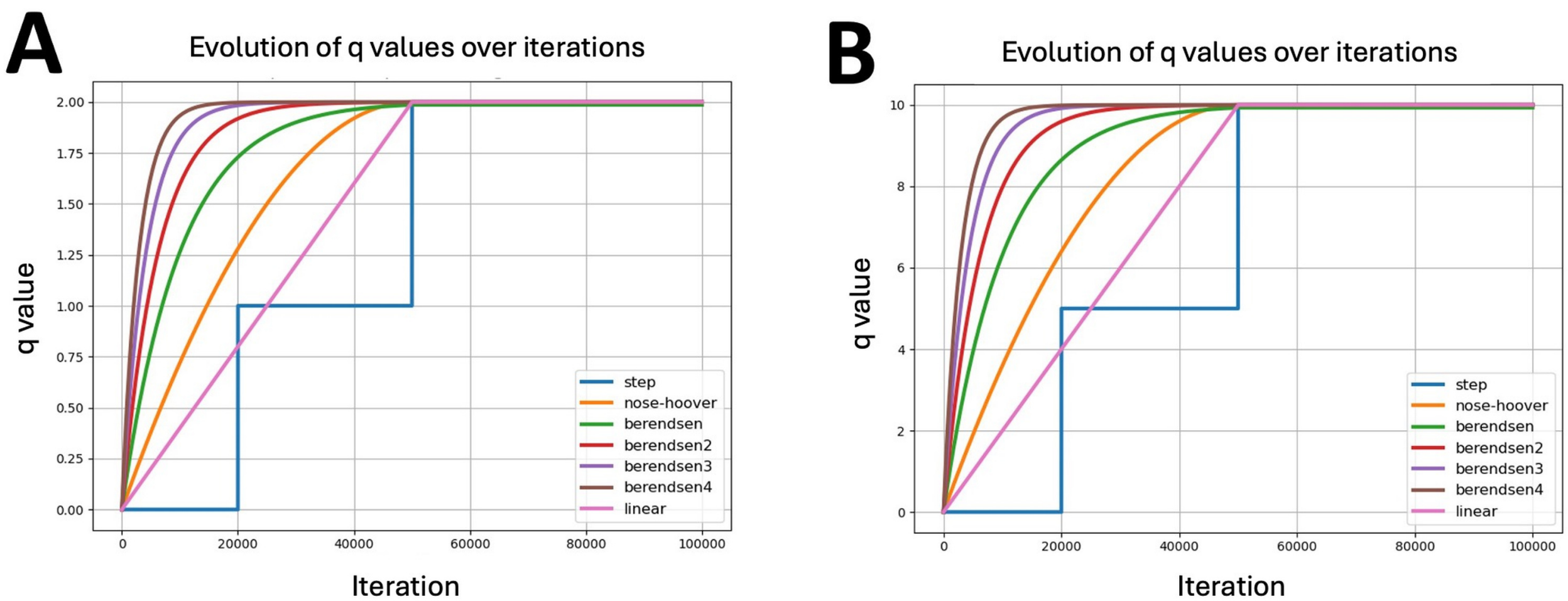

Figure S3. Comparison of the evolution of q values across q-scheduling methods. (A) Evolution of q values over iterations using various scheduling strategies with a target value of $q = 2$. (B) Same as in (A), but for $q = 10$. Berendsen-type methods show smoother convergence and outperform step scheduling.

All unknown kinetic parameters are optimized jointly with the network weights.

Physics-Informed Kolmogorov–Arnold Networks Architecture

To investigate whether structured representations improve inverse robustness, we replace the MLP with a Kolmogorov–Arnold Network (KAN) (17). KANs are motivated by the Kolmogorov–Arnold representation principle, which approximates multivariate functions via compositions of univariate nonlinear mappings rather than affine transformations.

In the Chebyshev-based variant (cPIKAN) (25, 26), the learnable univariate mappings are parameterized using Chebyshev polynomials:

$$\phi(\zeta) = \sum_{n=0}^{D} c_n T_n(\zeta), \tag{S14}$$

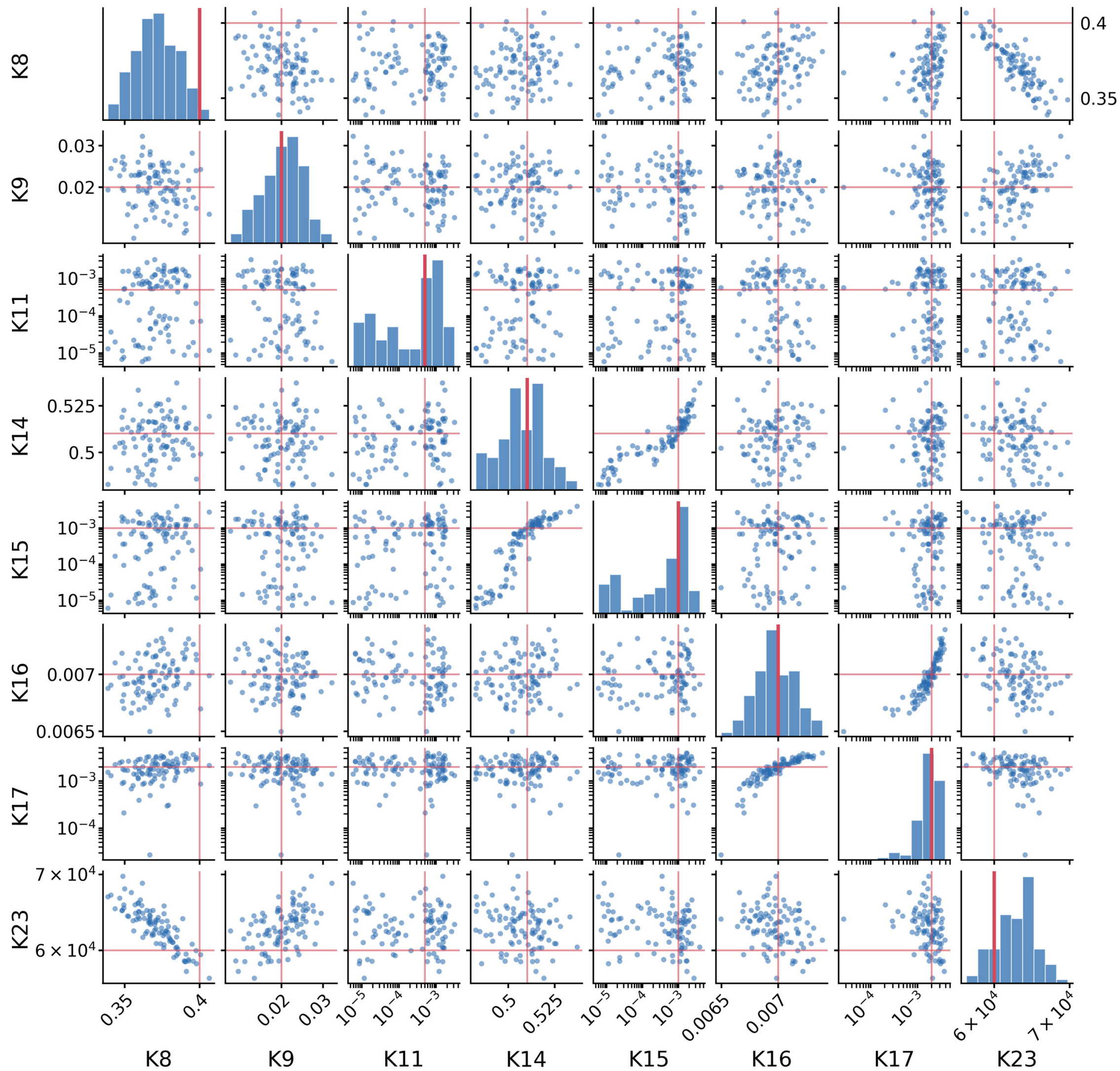

Figure S4. Distribution and pairwise correlation of the inferred kinetic parameters. Results of $N = 99$ independent Monte Carlo training runs of the parameters (K8, K9, K11, K14, K15, K16, K17, K23), each initialized with a different random seed (one further run failed to converge and was excluded). Diagonal panels show the marginal distribution of each parameter, with the ground-truth value marked by a red vertical line; off-diagonal panels show the pairwise joint distributions, with the ground-truth values of the two parameters marked by red lines whose intersection is the true parameter pair. Pearson correlation coefficients are computed in $\log_{10}$ space and the dominant ones are quoted in the text. The K_8 scale shared by the top row is given on the right-hand edge, the left-hand panel of that row being the histogram. All axes are logarithmic, consistent with the multiplicative parameterization $K = \exp(\theta)$ used during training, and are given in the absolute units of Table S2: s^{-1} for the rate constants and # (molecules per cell) for K_{23}.

where $T_n(\zeta)$ denotes the n-th Chebyshev polynomial and $\{c_n\}$ are trainable coefficients.

In this work, we adopt a modified architecture referred to as *tanh-cPIKAN*, which introduces nested tanh nonlinearities to improve numerical stability. Let $\sigma(x) = \tanh(x)$. For an L-layer

network, the forward mapping is defined recursively as

$$\mathbf{z}^{(0)} = \sigma(t), \tag{S15}$$

$$\mathbf{z}^{(\ell)} = \sigma\left(\sum_{i=1}^{H_\ell}\sum_{n=0}^{D} c_{i,n}^{(\ell)} T_n\Big(\sigma\big(z_i^{(\ell-1)}\big)\Big)\right), \quad \ell = 1, \ldots, L, \tag{S16}$$

with final linear readout

$$\hat{\mathbf{X}}(t) = W\mathbf{z}^{(L)}. \tag{S17}$$

The linear readout allows output scaling via W while preserving bounded intermediate representations throughout the network. Beyond bounding activations, the tanh nonlinearity modifies the optimization geometry. Let $\mathbf{z}(\theta)$ denote the Chebyshev expansion and define the contracted representation

$$\tilde{\mathbf{z}} = \tanh(\mathbf{z}).$$

By the chain rule, the parameter Jacobian becomes

$$\frac{\partial \tilde{\mathbf{z}}}{\partial \theta} = \operatorname{diag}\big(1 - \tanh^2(\mathbf{z})\big)\frac{\partial \mathbf{z}}{\partial \theta}.$$

Thus, tanh inserts a diagonal contraction matrix with entries in $(0, 1]$ into the Jacobian.

For the squared residual loss $\mathcal{L}(\theta) = \|r(\theta)\|^2$, the dominant curvature term is the Gauss–Newton matrix $H_{GN} = J^\top J$. With tanh activations, this becomes

$$H_{GN} = J_0^\top \operatorname{diag}\big(1 - \tanh^2(\mathbf{z})\big)^2 J_0,$$

where J_0 denotes the Jacobian of the polynomial expansion without contraction.

Consequently, tanh rescales curvature directions and attenuates those associated with large-magnitude activations. In Chebyshev-based networks, high-degree polynomial interactions may amplify derivatives and induce sharp curvature in the loss landscape. The repeated contraction induced by tanh mitigates this effect by limiting activation growth and reducing curvature anisotropy, thereby improving numerical stability during physics-informed training (13). For first-order optimizers such as Adam, this contraction promotes more balanced gradient magnitudes and reduces oscillatory behavior caused by high-frequency components.

Both the PINNs and PIKANs architectures are trained using the identical composite physics-informed loss; only the functional representation differs.

Pairwise scatter plots of Monte Carlo parameter estimates

To evaluate the variability and correlations among trainable parameters, we conducted 99 independent Monte Carlo (MC) replicates of the parameter estimation procedure and visualized the results in a scatter matrix (Figure S4). Each replicate is initialized with an independent random seed, which sets both the realization of the 5% multiplicative measurement noise added to the synthetic observations and the random initialization of the trainable parameters; the ensemble therefore reflects the combined effect of measurement noise and optimization variability. Each off-diagonal panel displays the joint distribution between two parameters, and the diagonal panels display the marginal distribution of each parameter, with the ground-truth value marked in red. Because the network parameterizes the rate constants multiplicatively as $K = \exp(\theta)$, the axes and the reported correlation coefficients are evaluated on a logarithmic scale, which is the natural scale for these quantities.

The dominant couplings in the ensemble are a strong positive correlation between K14 and K15 ($r = +0.91$), a strong positive correlation between K16 and K17 ($r = +0.84$), and a strong negative correlation between K8 and K23 ($r = -0.84$); a moderate positive correlation is also observed between K9 and K23 ($r = +0.52$), while the remaining pairs show weaker or no clear dependency, reflecting partial independence. These couplings identify the parameter combinations that the available observations constrain only jointly, and they correspond to the near-degenerate directions identified in the Fisher Information Matrix (FIM)-based analysis. We note that the MC correlations are measured directly from the ensemble of trained models and, unlike the FIM-based estimates, do not rely on a local quadratic approximation of the loss surface; the two analyses are therefore complementary rather than equivalent.

The ensemble also makes the practical identifiability of the individual parameters directly visible. Four of the eight parameters (K8, K14, K16, K23) are recovered tightly, with interquartile ranges below 0.03 decades (a few percent), and K9 and K17 are recovered moderately well. By contrast, K11 and K15 span close to three decades across the ensemble, and in roughly one quarter of the runs (28/99 and 27/99, respectively) they collapse to below one tenth of their true value while the remaining parameters and the fitted trajectories are essentially unaffected. These two parameters are therefore only weakly constrained by the available observations. This is consistent with the flat curvature directions identified in the FIM analysis and with the effect of the search limits reported in Table S7, where constraining the admissible range collapses the estimated variability of K_{11} by nearly two orders of magnitude. The strong K14–K15 correlation further indicates that it is largely the combination of these parameters, rather than K15 alone, that the data determine. We report these values with the corresponding caveat.

Effect of Collocation Strategy: Fixed vs. Random Sampling

To further evaluate the training robustness and sensitivity to the collocation point configuration, we analyzed the performance of both PINNs and Tanh-cPIKANs using two types of physics-based residual sampling strategies: (i) **fixed collocation points** (denoted by **F**), and (ii) **randomly resampled collocation points during training** (denoted by **R**). Here, F100 refers to training with 100 fixed collocation points, while R1000 denotes resampling 1000 collocation points at each epoch. The relative parameter estimation errors for PINNs and Tanh-cPIKANs across these configurations are summarized in Tables S10 and S11, respectively.

Our findings reveal several important trends:

(1) Increasing the number of collocation points does not guarantee improved performance. In both PINNs and Tanh-cPIKANs, increasing the number of fixed collocation points beyond a certain threshold (e.g., F1000 or F1500) does not lead to consistently lower parameter inference error. For some parameters, performance plateaus or even worsens. It highlights the diminishing returns and potential over-constraining effect of excessive residual points.

(2) Random resampling can improve generalization at low point counts, but its benefits diminish with more collocation points. Comparing R100 and R1000 to their fixed counterparts (F100 and F1000), we observe that random resampling often leads to lower variance and better accuracy at lower point counts. However, as the number of collocation points increases, the advantage of stochastic sampling reduces, and in some cases the performance becomes comparable or slightly inferior to fixed strategies. This suggests that the benefits of randomization are more pronounced in lower number of collocation points.

(3) Tanh-cPIKANs exhibit greater robustness across collocation strategies, particularly under noise and limited residual supervision. Regardless of whether fixed or random sampling is used, and regardless of the number of collocation points, Tanh-cPIKANs achieve lower

Table S10. PINNs: Relative parameter estimation errors (%) under fixed (F) and randomly resampled (R) collocation point configurations. F-N denotes training with N fixed collocation points, while R-N denotes resampling N points at each epoch. Errors are computed relative to nominal parameter values after convergence. Entries in **bold** indicate the most accurate configuration (lowest relative error) for each parameter across sampling strategies and Network architecture.

Params	F-100	F-200	F-1000	F-1500	R-100	R-200	R-1000	R-1500
K_8	17.87	18.36	7.82	6.96	22.51	14.52	**2.69**	4.14
K_9	3.86	5.82	1.86	1.35	5.12	2.26	1.36	1.76
K_{11}	6.70	5.36	**0.76**	2.60	4.32	2.81	1.73	3.14
K_{14}	0.46	0.27	0.05	0.07	0.65	0.13	0.09	0.04
K_{15}	1.73	0.59	**0.18**	0.21	1.03	0.55	0.24	0.40
K_{16}	4.48	2.60	1.99	1.75	**0.32**	3.97	2.15	1.76
K_{17}	6.73	4.54	2.65	2.00	**0.67**	0.75	2.66	1.95
K_{23}	3.26	3.15	2.43	2.20	2.97	1.55	1.84	2.78

Table S11. Tanh-cPIKANs: Relative parameter estimation errors (%) under fixed (F) and randomly resampled (R) collocation point configurations. F-N denotes training with N fixed collocation points, while R-N denotes resampling N points at each epoch. Errors are computed relative to nominal parameter values after convergence. Entries in **bold** indicate the most accurate configuration (lowest relative error) for each parameter across sampling strategies and Network architecture.

Params	F-100	F-200	F-1000	F-1500	R-100	R-200	R-1000	R-1500
K_8	21.05	15.66	7.16	6.12	14.00	14.38	6.45	8.16
K_9	4.39	2.09	2.42	**1.33**	4.14	2.96	4.54	1.64
K_{11}	26.01	15.11	12.50	7.18	16.17	11.50	6.39	4.54
K_{14}	12.02	3.27	0.63	0.09	2.60	1.73	0.76	**0.01**
K_{15}	20.49	17.28	5.17	1.24	5.96	6.37	3.84	3.27
K_{16}	2.45	2.19	1.44	1.47	2.67	0.61	0.51	1.37
K_{17}	3.36	2.93	0.73	1.50	5.16	0.57	1.65	1.41
K_{23}	1.84	1.33	1.92	1.56	2.20	2.57	**1.17**	1.85

relative error in estimating most parameters. This robustness can be attributed to their hierarchical representation and localized basis structure, which allows for more flexible adaptation to the physical constraints even with limited or noisy residual supervision.

(4) PINNs are more sensitive to noise and sampling choices. In the PINNs results, several parameters—such as K_8, K_9, and K_{15}—exhibit error increases of up to fourfold when transitioning from 0% to 10% noise, and training performance varies significantly across collocation strategies. In contrast, Tanh-cPIKANs maintain stable performance under the same conditions.

(5) Random sampling does not uniformly outperform fixed sampling. Although random sampling has theoretical benefits in reducing overfitting to specific collocation locations and improving generalization, our experiments show that this is not universally the case. For both PINNs and PIKANs, certain parameters (e.g., K_{14}, K_{16}) show minimal sensitivity to the sampling method, while others show fluctuating gains. This indicates that the effectiveness of random sampling is model- and problem-dependent. In summary, these results highlight the nuanced trade-offs in collocation point strategies. While increasing collocation points or using random resampling can help under certain conditions, these factors alone are not sufficient to guarantee improved parameter recovery. Instead, architectural choices—such as those employed in tanh-cPIKANs—play a more critical role in ensuring robustness and accuracy in inverse problem settings.

SI References

[1] Herman JC Berendsen, JPM van Postma, Wilfred F Van Gunsteren, ARHJ DiNola, and Jan R Haak. Molecular dynamics with coupling to an external bath. *The Journal of Chemical Physics*, 81(8):3684–3690, 1984.

[2] Zhaojie Chai, Shiju Gu, and George Lykotrafitis. Dynamics of the axon plasma membrane skeleton. *Soft Matter*, 19(14):2514–2528, 2023.

[3] Zhaojie Chai, Anastasios V Tzingounis, and George Lykotrafitis. The periodic axon membrane skeleton leads to Na nanodomains but does not impact action potentials. *Biophysical Journal*, 121(18):3334–3344, 2022.

[4] Mitchell Daneker, Zhen Zhang, George Em Karniadakis, and Lu Lu. Systems biology: Identifiability analysis and parameter identification via systems-biology-informed neural networks. In *Computational Modeling of Signaling Networks*, pages 87–105. Springer, 2023.

[5] Pep Espanol and Patrick Warren. Statistical mechanics of dissipative particle dynamics. *Europhysics Letters*, 30(4):191–196, 1995.

[6] Pep Espanol and Patrick B Warren. Perspective: Dissipative particle dynamics. *The Journal of Chemical Physics*, 146(15), 2017.

[7] D.A. Fedosov, B. Caswell, and G.E. Karniadakis. A multiscale red blood cell model with accurate mechanics, rheology, and dynamics. *Biophysical Journal*, 98(10):2215–2225, 2010.

[8] Karl Francis and Bernhard O Palsson. Effective intercellular communication distances are determined by the relative time constants for cyto/chemokine secretion and diffusion. *Proceedings of the National Academy of Sciences*, 94(23):12258–12262, 1997.

[9] Robert D Groot and Patrick B Warren. Dissipative particle dynamics: Bridging the gap between atomistic and mesoscopic simulation. *The Journal of Chemical Physics*, 107(11):4423–4435, 1997.

[10] PJ Hoogerbrugge and JMVA Koelman. Simulating microscopic hydrodynamic phenomena with dissipative particle dynamics. *Europhysics Letters*, 19(3):155–160, 1992.

[11] George Em Karniadakis, Ioannis G Kevrekidis, Lu Lu, Paris Perdikaris, Sifan Wang, and Liu Yang. Physics-informed machine learning. *Nature Reviews Physics*, 3(6):422–440, 2021.

[12] Sanjay Khandelwal, Nico Van Rooijen, and Rajiv K Saxena. Reduced expression of CD47 during murine red blood cell (RBC) senescence and its role in RBC clearance from the circulation. *Transfusion*, 47(9):1725–1732, 2007.

[13] Siavash Khodakarami, Vivek Oommen, Nazanin Ahmadi Daryakenari, Maxim Beekenkamp, and George Em Karniadakis. Spectral bias in physics-informed and operator learning: Analysis and mitigation guidelines. *arXiv preprint arXiv:2602.19265*, 2026.

[14] Huan Lei, Bruce Caswell, and George Em Karniadakis. Direct construction of mesoscopic models from microscopic simulations. *Physical Review E*, 81(2):026704, 2010.

[15] He Li and George Lykotrafitis. Erythrocyte membrane model with explicit description of the lipid bilayer and the spectrin network. *Biophysical Journal*, 107(3):642–653, 2014.

[16] He Li, Yihao Zhang, Vi Ha, and George Lykotrafitis. Modeling of band-3 protein diffusion in the normal and defective red blood cell membrane. *Soft Matter*, 12(15):3643–3653, 2016.

[17] Ziming Liu, Yixuan Wang, Sachin Vaidya, Fabian Ruehle, James Halverson, Marin Soljačić, Thomas Y Hou, and Max Tegmark. KAN: Kolmogorov-Arnold networks. *arXiv preprint arXiv:2404.19756*, 2024.

[18] Simone Monaco and Daniele Apiletti. Training physics-informed neural networks: One learning to rule them all? *Results in Engineering*, 18:101023, 2023.

[19] Shuichi Nosé. A unified formulation of the constant temperature molecular dynamics methods. *The Journal of Chemical Physics*, 81(1):511–519, 1984.

[20] Manoj K Pandey, Thomas A Burrow, Reena Rani, Lisa J Martin, David Witte, Kenneth D Setchell, Mary A Mckay, Albert F Magnusen, Wujuan Zhang, Benjamin Liou, et al. Complement drives glucosylceramide accumulation and tissue inflammation in Gaucher disease. *Nature*, 543(7643):108–112, 2017.

[21] Biserka Pokrić and Zvonimir Pučar. The two-cross immunodiffusion technique: diffusion coefficients and precipitating titers of IgG in human serum and rabbit serum antibodies. *Analytical Biochemistry*, 93:103–114, 1979.

[22] Tailong Qu, Baiyong Li, and Yifei Wang. Targeting CD47/SIRPα as a therapeutic strategy, where we are and where we are headed. *Biomarker Research*, 10(1):20, 2022.

[23] A. Rajabi, E. Javadi, S. R. Pejman Sereshkeh, M. R. Morad, A. Kebriaee, H. Nasiri, and S. A. A. Razavi Haeri. Experimental characterization of an extended electrohydrodynamic cone-jet with a hemispherical nozzle. *Physics of Fluids*, 30(11):114108, 11 2018.

[24] Andreas Raue, Clemens Kreutz, Thomas Maiwald, Julie Bachmann, Marcel Schilling, Ursula Klingmüller, and Jens Timmer. Structural and practical identifiability analysis of partially observed dynamical models by exploiting the profile likelihood. *Bioinformatics*, 25(15):1923–1929, 2009.

[25] Khemraj Shukla, Juan Diego Toscano, Zhicheng Wang, Zongren Zou, and George Em Karniadakis. A comprehensive and FAIR comparison between MLP and KAN representations for differential equations and operator networks. *Computer Methods in Applied Mechanics and Engineering*, 431:117290, 2024.

[26] Sidharth SS, Keerthana AR, Anas KP, et al. Chebyshev polynomial-based Kolmogorov-Arnold networks: An efficient architecture for nonlinear function approximation. *arXiv preprint arXiv:2405.07200*, 2024.

[27] Yu-Hang Tang, Lu Lu, He Li, Constantinos Evangelinos, Leopold Grinberg, Vipin Sachdeva, and George Em Karniadakis. OpenRBC: A fast simulator of red blood cells at protein resolution. *Biophysical Journal*, 112(10):2030–2037, 2017.

[28] Juan Diego Toscano, Zhaojie Chai, and George Em Karniadakis. GRAFT-ATHENA: Self-improving agentic teams for autonomous discovery and evolutionary numerical algorithms. *arXiv preprint arXiv:2605.11117*, 2026.

[29] Rajat Varma and Satyajit Mayor. GPI-anchored proteins are organized in submicron domains at the cell surface. *Nature*, 394(6695):798–801, 1998.

[30] André Veillette and Jun Chen. SIRPα–CD47 immune checkpoint blockade in anticancer therapy. *Trends in Immunology*, 39(3):173–184, 2018.

[31] Sifan Wang, Yujun Teng, and Paris Perdikaris. Understanding and mitigating gradient flow pathologies in physics-informed neural networks. *SIAM Journal on Scientific Computing*, 43(5):A3055–A3081, 2021.

[32] Franz-Georg Wieland, Adrian L Hauber, Marcus Rosenblatt, Christian Tönsing, and Jens Timmer. On structural and practical identifiability. *Current Opinion in Systems Biology*, 25:60–69, 2021.

[33] Daniel J Wolak, Michelle E Pizzo, and Robert G Thorne. Probing the extracellular diffusion of antibodies in brain using in vivo integrative optical imaging and ex vivo fluorescence imaging. *Journal of Controlled Release*, 197:78–86, 2015.

[34] Chenxi Wu, Min Zhu, Qinyang Tan, Yadhu Kartha, and Lu Lu. A comprehensive study of non-adaptive and residual-based adaptive sampling for physics-informed neural networks. *Computer Methods in Applied Mechanics and Engineering*, 403:115671, 2023.

[35] Zhen-Hua Wu, Na Li, Xiao-Feng Mei, Juan Chen, Xiao-Ze Wang, Ting-Ting Guo, Gang Chen, Lei Nie, Yao Chen, Mei-Zhu Jiang, et al. Preclinical characterization of the novel anti-SIRPα antibody BR105 that targets the myeloid immune checkpoint. *Journal for Immunotherapy of Cancer*, 10(3):e004054, 2022.

[36] Zixue Xiang, Wei Peng, Xu Liu, and Wen Yao. Self-adaptive loss balanced physics-informed neural networks. *Neurocomputing*, 496:11–34, 2022.

[37] Alireza Yazdani, Lu Lu, Maziar Raissi, and George Em Karniadakis. Systems biology informed deep learning for inferring parameters and hidden dynamics. *PLOS Computational Biology*, 16(11):e1007575, 2020.

[38] Yihao Zhang, Zhaojie Chai, and George Lykotrafitis. Deep reinforcement learning with a particle dynamics environment applied to emergency evacuation of a room with obstacles. *Physica A: Statistical Mechanics and its Applications*, 571:125845, 2021.

[39] Yihao Zhang, Zhaojie Chai, Yubing Sun, and George Lykotrafitis. A deep reinforcement learning model based on deterministic policy gradient for collective neural crest cell migration. *arXiv preprint arXiv:2007.03190*, 2020.

[40] Yihao Zhang, Anastasios V Tzingounis, and George Lykotrafitis. Modeling of the axon plasma membrane structure and its effects on protein diffusion. *PLOS Computational Biology*, 15(5):e1007003, 2019.

[41] Yu Zhang, Yuhao Qiang, He Li, Guansheng Li, Lu Lu, Ming Dao, George E Karniadakis, Aleksander S Popel, and Chen Zhao. Signaling-biophysical modeling unravels mechanistic control of red blood cell phagocytosis by macrophages in sickle cell disease. *PNAS Nexus*, 3(2):pgae031, 2024.
